# SHMT2 deficiency disrupts transcriptional regulation through homocysteine-mediated suppression of histone lactylation in Huntington’s disease models

**DOI:** 10.1172/JCI196094

**Published:** 2026-03-10

**Authors:** Mingqin Lu, Kexin Li, Shanshan Wu, Zhilong Zheng, Xinyue Li, Shengda Wang, Hanwen Yu, Chunyue Liu, Yueqing Jiang, Xueqin Song, Yan Liu, Xing Guo

**Affiliations:** 1 Department of Neurobiology, School of Basic Medical Sciences,; 2 State Key Laboratory of Reproductive Medicine and Offspring Health, and; 3 Institute for Stem Cell and Neural Regeneration, School of Pharmacy, Nanjing Medical University, Nanjing, Jiangsu, China.; 4 Key Laboratory of Clinical Neurology, Ministry of Education, Hebei Medical University, Shijiazhuang, Hebei, China.; 5 Department of Neurology, Key Neurological Laboratory of Hebei Province, the Second Hospital of Hebei Medical University, Shijiazhuang, Hebei, China.; 6 Jiangsu Key Laboratory of Molecular Targets and Intervention for Metabolic Diseases, Nanjing Medical University, Nanjing, Jiangsu, China.

**Keywords:** Aging, Cell biology, Neuroscience, Neurodegeneration

## Abstract

Huntington’s disease (HD) is a fatal neurodegenerative disorder characterized by progressive motor dysfunction, cognitive decline, and striatal neuron degeneration, primarily affecting medium spiny neurons (MSNs). Despite extensive research, the underlying metabolic vulnerabilities contributing to HD pathogenesis remain poorly understood. In this study, we employed RNA-seq and metabolomics analyses to identify marked dysregulation of 1-carbon metabolism in HD. We validated that SHMT2, a key mitochondrial enzyme in the mitochondrial 1-carbon pathway, was substantially downregulated in HD patient–derived iPSC-differentiated human striatal organoids (hSOs) and YAC128 mice. Functionally, pharmacologic inhibition or genetic deletion of SHMT2 exacerbated mutant huntingtin aggregation, induced MSN degeneration in hSOs, and impaired motor function in WT mice. Conversely, SHMT2 overexpression attenuated MSN degeneration in HD-hSOs and improved motor performance in YAC128 mice. Mechanistically, SHMT2 deficiency led to accumulation of homocysteine, which interacted with AARS1 and suppressed histone lactylation, thereby perturbing transcriptional regulation and associating with neurodegenerative phenotypes. Finally, we demonstrated that the HD clinical drug haloperidol modulated SHMT2 expression and restored histone lactylation, providing a pharmacologic tool to probe SHMT2-dependent metabolic and epigenetic regulation in HD models. These findings highlight a metabolic-epigenetic axis as a promising therapeutic target for HD.

## Abstract 2

SHMT2 deficiency elevates homocysteine, which suppresses histone lactylation and perturbs transcriptional regulation associated with neurodegenerative phenotypes in Huntington’s disease models.

## Graphical abstract

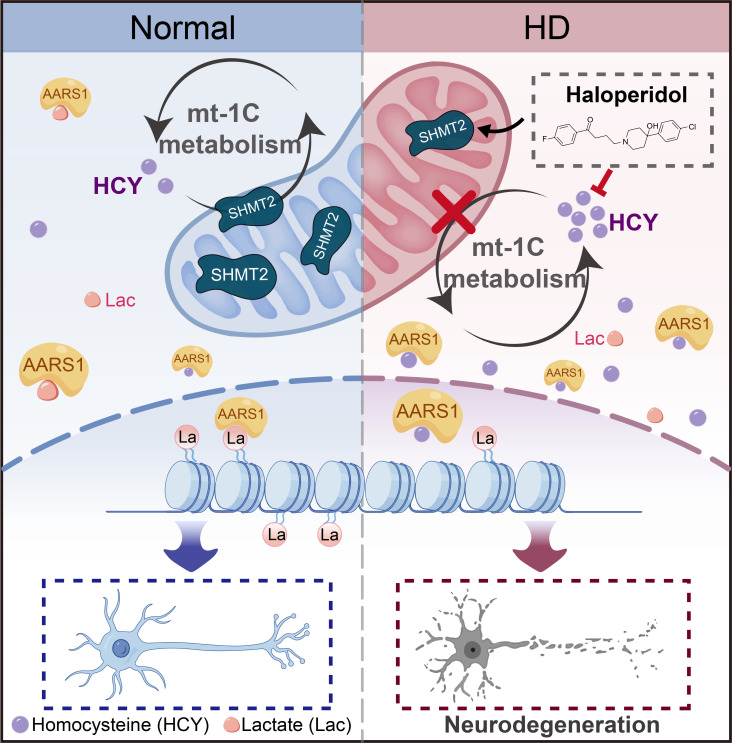

## Introduction

Huntington’s disease (HD) is a fatal autosomal dominant neurodegenerative disorder caused by a CAG repeat expansion in the *HTT* gene (1, 2). HD manifests as a progressive neurodegenerative disorder marked by motor impairments, cognitive deterioration, and selective loss of striatal neurons, particularly medium spiny neurons (MSNs), which play a crucial role in motor control and basal ganglia circuitry (3–5). Although considerable progress has been made in understanding HD pathology, the lack of effective disease-modifying therapies underscores the need to further explore the molecular and metabolic mechanisms underlying neurodegeneration.

Mitochondrial 1-carbon (mt-1C) metabolism is a fundamental biochemical pathway that supports nucleotide synthesis, epigenetic regulation (DNA/RNA methylation), redox homeostasis, and mitochondrial energy production (6, 7). Given its essential role in maintaining neuronal function and genomic stability, disruptions in mt-1C metabolism have been implicated in neurodegenerative diseases (8, 9). In Alzheimer’s disease (AD), impaired folate cycle activity leads to abnormal DNA methylation patterns and increased oxidative stress, contributing to cognitive decline (10). Similarly, in Parkinson’s disease (PD), dysregulated methionine metabolism and redox imbalance are associated with mitochondrial dysfunction and dopaminergic neuron degeneration (11). However, the involvement of mt-1C metabolism in HD has not been systematically investigated.

A key regulator of mt-1C metabolism is serine hydroxymethyltransferase 2 (SHMT2), a mitochondrial enzyme that catalyzes the conversion of serine to glycine, supplying 1-carbon (1C) units to the tetrahydrofolate (THF) cycle (12–15). SHMT2 dysfunction disrupts nucleotide biosynthesis, redox balance, and epigenetic regulation, which are essential for neuronal survival, and has been implicated in metabolic and mitochondrial abnormalities across various human diseases, including cancer and cardiovascular disorders (16–18). Given its central metabolic role and neuronal relevance, SHMT2 may serve as a key node linking mitochondrial dysfunction to neurodegeneration in HD.

Recent studies suggest that homocysteine (HCY), a key intermediate in the methionine cycle, is metabolically coupled to mt-1C metabolism through folate-mediated remethylation (19). Elevated HCY levels contribute to multiple neurotoxic effects, including oxidative stress, mitochondrial dysfunction, excitotoxicity, and DNA methylation abnormalities (20–22). In neurodegenerative diseases such as AD and PD, HCY accumulation has been associated with pathological features including tau hyperphosphorylation, amyloid-β aggregation, disruption of redox balance, and inhibition of mitochondrial complex I activity, all of which exacerbate neuronal damage (23, 24). Moreover, HCY interferes with S-adenosylmethionine (SAM) metabolism, limiting methyl donor availability and resulting in global DNA hypomethylation and transcriptional abnormalities, which are hallmarks of neurodegeneration (25).

In this study, we identified 1C metabolic dysfunction as a key contributor to HD pathogenesis. We demonstrate that SHMT2, a central mt-1C enzyme, is consistently downregulated in HD models, resulting in HCY accumulation and reduced histone lactylation, which together exacerbate neuronal damage and drive neurodegeneration. Mechanistically, elevated HCY interacts with AARS1, further perturbing histone lactylation and associating with adverse neuronal outcomes. Restoring SHMT2 expression rescues histone lactylation, reduces neuronal loss, and improves functional outcomes. Notably, haloperidol was used as a pharmacological probe to perturb the SHMT2-HCY-lactylation axis, and it partially restored SHMT2 expression, reduced HCY levels, and enhanced histone lactylation, providing mechanistic insight into this pathway. Together, these findings uncover what we believe to be a previously unrecognized metabolic-epigenetic mechanism in HD and highlight SHMT2 as a potential therapeutic target.

## Results

### Multi-omics analysis reveals 1C metabolism dysregulation and reduced SHMT2 expression in HD models.

**Figure 1:**
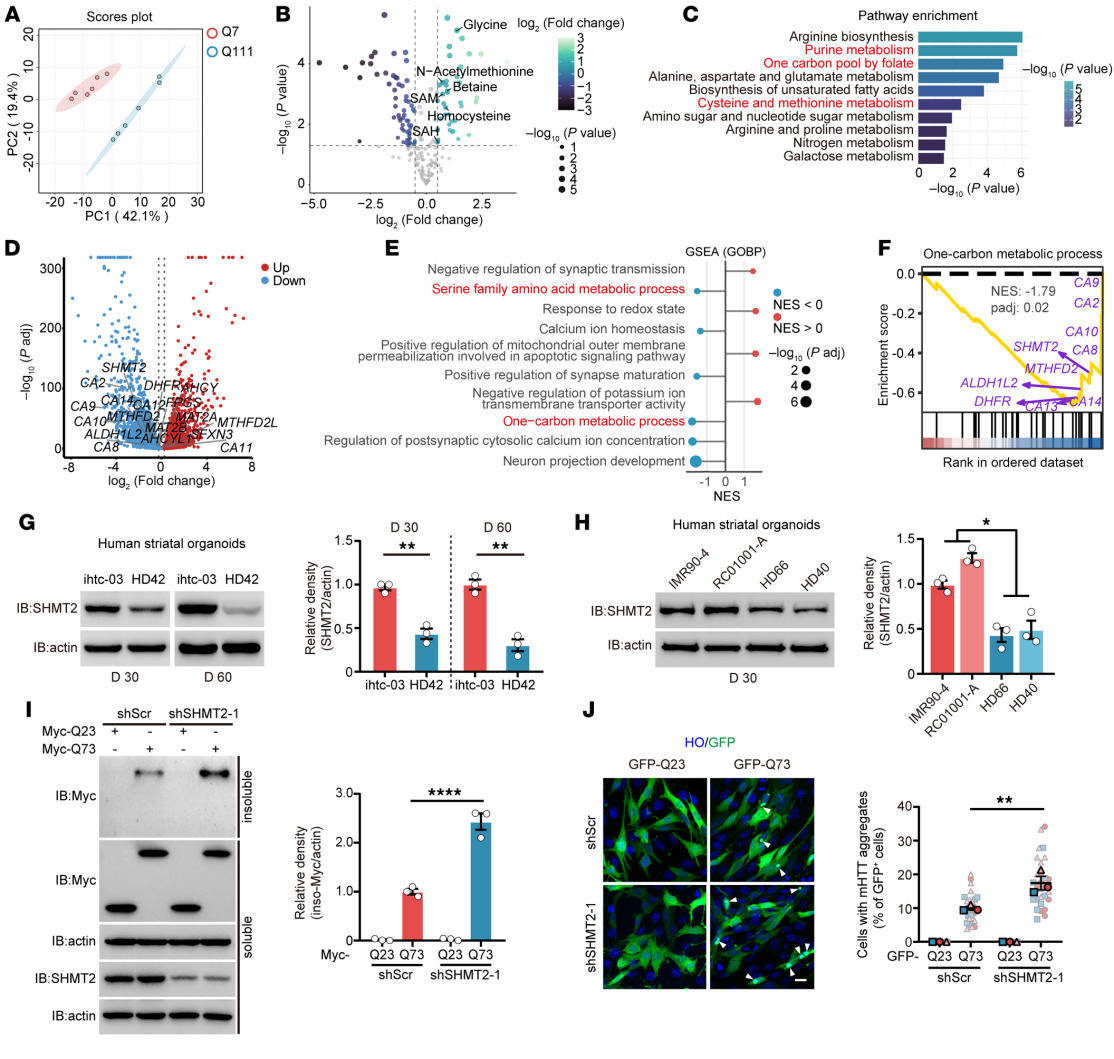
Multi-omics analysis reveals 1C metabolism dysregulation and reduced SHMT2 expression in HD models. (**A**) Principal component analysis (PCA) of metabolomic profiles in HdhQ7 and HdhQ111 cells. (**B**) Volcano plot showing substantially altered metabolites in HdhQ111 cells relative to HdhQ7 cells. One-carbon metabolism–related metabolites are highlighted. (**C**) Pathway enrichment analysis of markedly altered metabolites between HdhQ7 and HdhQ111 cells. (**D**) Volcano plot of DEGs in HD-hSOs (HD42) versus control hSOs (RC01001-A), showing widespread transcriptional alterations. (**E**) GSEA (GO Biological Process) showing pathway changes in HD-hSOs. (**F**) GSEA enrichment plot for the 1-carbon metabolic process gene set. (**G**) Immunoblot analysis of SHMT2 protein levels in hSOs derived from HD patient iPSCs (HD42) and iPSCs from a control line (ihtc-03) at D30 and D60 (*n* = 3). (**H**) Immunoblot analysis of SHMT2 protein levels in additional hSOs derived from HD patient iPSCs (HD66 and HD40) and control iPSCs (IMR90-4 and RC01001-A) at D30 (*n* = 3). (**I**) Soluble and insoluble HTTEx1-Q23/Q73 in control and SHMT2-knockdown HdhQ7 cells transfected with Myc-tagged HTTEx1-Q23 or HTTEx1-Q73 for 48 hours (*n* = 3). (**J**) Representative immunofluorescence images and quantification of polyQ aggregates in control and SHMT2-knockdown HdhQ7 cells transfected with GFP-tagged HTTEx1-Q23 or HTTEx1-Q73. Arrows indicate visible aggregates (scale bar: 20 μm; *n* = 3). Data are shown as the mean ± SEM. Unpaired *t* test was used for **G**, nested *t* test was used for **H**, and 1-way ANOVA with Tukey’s test was used for **I** and **J**. *P < 0.05, **P < 0.01, ****P < 0.0001.

To analyze metabolic alterations in HD, we performed targeted metabolomics analysis in HdhQ7 and HdhQ111 cells (Figure 1A). We quantified 256 metabolites in these cells (Supplemental Table 1; supplemental material available online with this article; https://doi.org/10.1172/JCI196094DS1). Compared with HdhQ7 cells, 104 metabolites exhibited notable alterations in HdhQ111 cells (Figure 1B). Notably, metabolites associated with 1C metabolism, including HCY, SAM, S-adenosylhomocysteine, N-acetylmethionine, and glycine, were elevated (Figure 1B and Supplemental Figure 1A). Pathway enrichment analysis revealed notable alterations in several metabolic pathways associated with 1C metabolism, including cysteine and methionine metabolism, purine metabolism, and 1C pool by folate, indicating dysregulation of the 1C metabolic network in HD (Figure 1C). To further validate the transcriptional disruption of 1C metabolism, we performed RNA-seq in human induced pluripotent stem cell–derived (iPSC-derived) striatal organoids generated from a control line (RC01001-A) and an HD patient line (HD42) (Supplemental Figure 1B), using protocols established in our previous study (26). Volcano plot analysis revealed dysregulation of multiple 1C metabolic enzymes, including SHMT2, ALDH1L2, MTHFD2, MTHFD2L, and DHFR, as well as methionine cycle–related genes *MAT2B* and folate-metabolism-related gene *FPGS* (Figure 1D and Supplemental Table 2). GSEA further showed that the 1C metabolic process was notably downregulated in human striatal organoids (hSOs) derived from patients with HD (HD-hSOs), accompanied by alterations in synaptic, calcium-homeostasis, and redox-related pathways (Figure 1, E and F). A parallel RNA-seq analysis of HdhQ7 and HdhQ111 cells yielded consistent results, similarly highlighting coordinated dysregulation of key 1C metabolic enzymes (Supplemental Figure 1, C–F, and Supplemental Table 3), demonstrating that 1C metabolic dysfunction is a conserved feature across independent HD models.

SHMT2 is a central regulator of mt-1C metabolism and plays a crucial role in folate cycle regulation, nucleotide biosynthesis, and redox homeostasis. Its disruption impairs mitochondrial function, leading to metabolic imbalances and increased oxidative stress. We therefore assessed SHMT2 abundance in day 30 (D30) and D60 hSOs derived from a control line (ihtc-03) and HD42, and found a marked decrease in HD-hSOs (Figure 1G). Furthermore, this reduction was also observed in hSOs generated from additional control (IMR90-4, RC01001-A) and HD (HD66, HD40) iPSC line and was independently confirmed in hSO-derived neurons from 3 control and 3 HD lines, supporting the robustness of this finding (Figure 1H and Supplemental Figure 1G). Additionally, we confirmed the reduction of SHMT2 levels in multiple HD models, including HdhQ111 cells and YAC128 mice, compared with their respective controls (Supplemental Figure 1, H and I). Western blot analysis showed an increase in insoluble polyQ protein levels in SHMT2-deficient cells, while immunostaining revealed a corresponding rise in the number of cells with polyQ aggregates (Figure 1, I and J, and Supplemental Figure 1, J and K). Together, these findings demonstrate that 1C metabolism is dysregulated in HD, with SHMT2 downregulation associated with increased polyQ aggregate accumulation.

### Loss of SHMT2 induces neuronal degeneration in iPSCs-derived hSOs.

**Figure 2:**
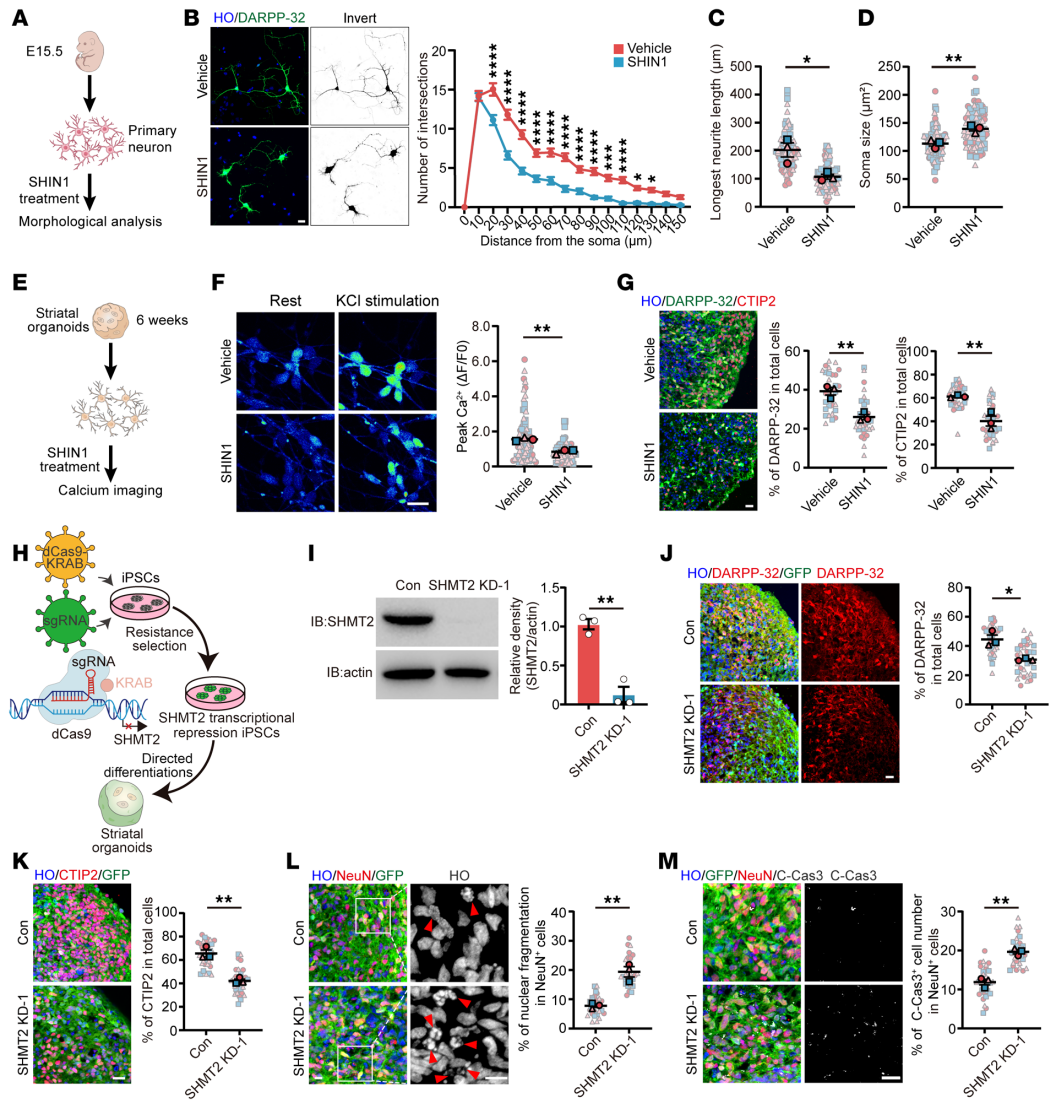
Loss of SHMT2 induces neuronal degeneration in iPSCs-derived hSOs. (**A** and **B**) Schematic of SHIN1 treatment in primary striatal neurons and Sholl analysis of neuronal complexity after 48 hours of SHIN1 (10 μM) or DMSO at DIV3 (scale bar: 10 μm; *n* = 60). (**C** and **D**) Quantification of longest neurite length and soma size in primary striatal neurons treated for 48 hours with SHIN1 (10 μM) or DMSO at DIV3 (*n* = 3). (**E** and **F**) Schematic of SHIN1 treatment in hSO-derived neurons and calcium imaging showing baseline and KCl–evoked (67 mM) responses, with quantification of peak Ca^2+^ signals after 48 hours of DMSO or SHIN1 treatment (scale bar: 20 μm; *n* = 3). (**G**) DARPP-32 (green) and CTIP2 (red) staining in D60 hSOs after 7 days of SHIN1 treatment (scale bar: 20 μm; *n* = 3). (**H** and **I**) Schematic of CRISPRi-mediated SHMT2 knockdown in iPSCs and immunoblot validation of SHMT2 reduction in hSOs derived from sgRNA-transduced iPSCs (*n* = 3). (**J** and **K**) Representative images of DARPP-32/GFP and CTIP2/GFP costaining in control and SHMT2-knockdown hSOs at D60, with quantification of DARPP-32^+^ and CTIP2^+^ cell proportions (scale bar: 20 μm; *n* = 3). (**L**) Nuclear fragmentation in NeuN^+^ neurons from control and SHMT2-knockdown hSOs at D60, with quantification of fragmented nuclei (scale bar: 10 μm; *n* = 3). (**M**) Cleaved caspase-3 staining in NeuN^+^ neurons from control and SHMT2-deficient hSOs at D60, with quantification of cleaved caspase-3^+^ neurons (scale bar: 20 μm; *n* = 3). Data are shown as the mean ± SEM. Two-way ANOVA with Šídák’s test was used for **B**; unpaired *t* test for **C**, **D**, **F**, **G**, and **I**–**M**. *P < 0.05, **P < 0.01, ***P < 0.001, ****P < 0.0001.

To elucidate the function of SHMT2 in the MSNs, we first isolated primary MSNs from the striatum of WT mice (Figure 2A). The cultured neurons were treated with SHIN1, a potent inhibitor targeting SHMT isoforms, which are essential for 1C metabolism and catalyze the conversion of serine to glycine (27). Inhibition of SHMT decreased neurite complexity, as indicated by a reduced number of intersections among microstructured networks and a shortened longest neurite length (Figure 2, B and C). In addition, neuronal soma size was increased, suggesting potential alterations in cellular structure or homeostatic regulation (Figure 2D). To assess neuronal function, we measured calcium signaling in neurons derived from hSOs and found that SHMT inhibition substantially reduced the peak Ca^2+^ response to KCl stimulation (Figure 2, E and F). Furthermore, we analyzed DARPP-32^+^ and CTIP2^+^ neuronal populations, key markers of MSNs, and found that their proportions were markedly reduced in hSOs treated with SHIN1 (Figure 2G), along with increased cleaved-caspase-3^+^ apoptosis in DARPP-32^+^ neurons (Supplemental Figure 2A). We next generated iPSC lines with reduced SHMT2 expression using CRISPR interference (CRISPRi) technology (Figure 2H). Using 2 independent sgRNAs, SHMT2 knockdown reduced the proportions of DARPP-32^+^ MSNs and CTIP2^+^ neurons within hSOs, with single-cell analysis confirming a concordant decrease specifically in the DARPP-32^+^ MSN population and also impaired neurite complexity, as assessed by Sholl analysis (Figure 2, I–K, and Supplemental Figure 2, B–F). Importantly, SHMT2 deletion led to robust neuronal apoptosis, with elevated cleaved caspase-3 and fragmented nuclei observed in NeuN^+^ neurons in hSOs and in dissociated DARPP-32^+^ neurons (Figure 2, L and M, and Supplemental Figure 2, G–I). Together, these results demonstrate that SHMT2 is essential for maintaining MSN structural integrity, functional activity, and survival in hSOs.

### SHMT2 deficiency induces neurodegeneration and motor dysfunction in vivo.

**Figure 3:**
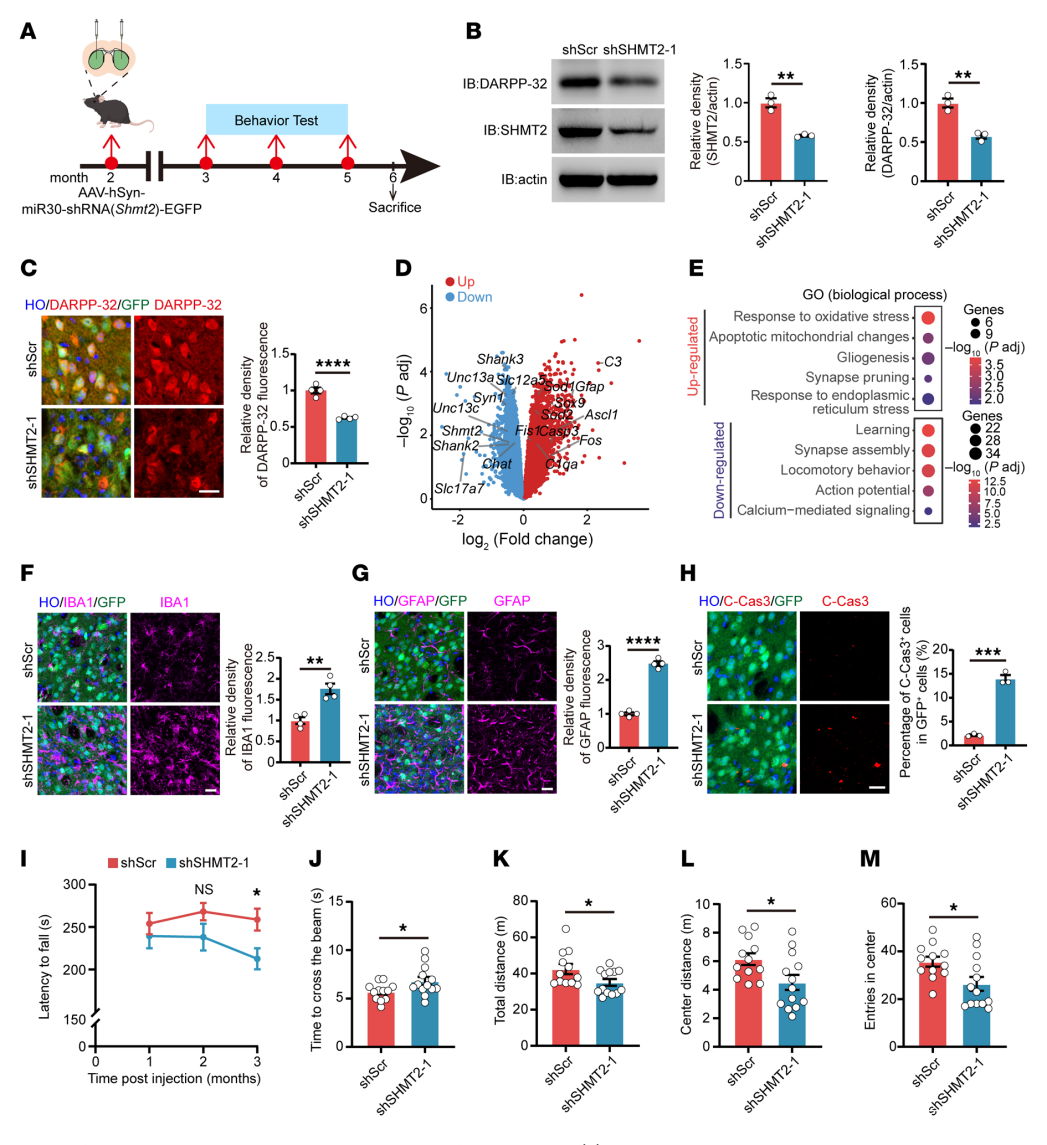
SHMT2 deficiency induces neurodegeneration and motor dysfunction in vivo. (**A**) Schematic timeline of stereotaxic injections of AAV-shScr or AAV-shSHMT2 into the striatum of 2-month-old mice, followed by behavioral and pathological analyses at indicated time points. (**B**) SHMT2 and DARPP-32 protein levels in the striatum of AAV-shScr– and AAV-shSHMT2–injected mice (*n* = 3). (**C**) DARPP-32 (red) and GFP (green) staining in brain sections from AAV-shScr– and AAV-shSHMT2–injected mice. DARPP-32 fluorescence density is quantified within a 220 μm radius around the injection site (*n* = 4; scale bar: 20 μm). (**D**) Volcano plot showing markedly altered genes in striatal RNA-seq of SHMT2-knockdown mice compared with controls. DEG cutoff: |log_2_FC| ≥ 0.25, *P*_adj_ ≤ 0.05. (**E**) GO Biological Process enrichment of DEGs from striatal RNA-seq in SHMT2-knockdown versus control mice. Bubble size reflects gene number; color indicates –log_10_(*P*_adj_). (**F**) IBA1 (magenta) and GFP staining with fluorescence density quantified within a 220 μm radius around the injection site (*n* = 4; scale bar: 20 μm). (**G**) GFAP (magenta) and GFP (green) staining with fluorescence density quantified within a 220 μm radius around the injection site (*n* = 4; scale bar: 20 μm). (**H**) Cleaved caspase-3 (red) and GFP (green) in striatal sections; percentage of cleaved caspase-3^+^ cells among GFP^+^ cells quantified (*n* = 3; scale bar: 20 μm). (**I**–**M**) Behavioral analyses following AAV-shScr or AAV-shSHMT2 injection in 2-month-old mice (*n* = 12–13). (**I**) Rotarod performance measured monthly. (**J**) Beam-crossing time at 2 months after injection. (**K**–**M**) Open-field test at 3 months: total distance (**K**), center distance (**L**), and center entries (**M**). Data are shown as the mean ± SEM. Unpaired *t* test was used for **B**, **C**, and **F**–**M**. *P < 0.05, **P < 0.01, ***P < 0.001, ****P < 0.0001.

To further investigate the role of SHMT2 in vivo, we generated adeno-associated virus (AAV) vectors carrying shRNA targeting *Shmt2*. Two-month-old C57BL/6 mice underwent stereotactic injection of AAV-shSHMT2 or control AAV-shScr into the striatum, followed by behavioral testing and biochemical analysis (Figure 3A). In the striatum of AAV-shSHMT2–injected mice, SHMT2 knockdown resulted in a notable downregulation of DARPP-32, a key regulator of intracellular signaling, synaptic plasticity, and neuronal excitability in MSNs (Figure 3, B and C, and Supplemental Figure 3, A and B). To delineate the transcriptional changes induced by SHMT2 knockdown, we performed comparative RNA-seq on the striatum of SHMT2-knockdown mice and their control littermates. Gene Ontology analysis revealed prominent activation of pathways related to oxidative stress and mitochondrial injury, accompanied by suppression of neuronal functional programs related to synaptic organization, action potential firing, and calcium-mediated signaling (Figure 3, D and E, Supplemental Figure 3C, and Supplemental Table 4). Concurrently, the fluorescence density of GFAP^+^ astrocytes and IBA1^+^ microglia was clearly increased, indicative of reactive gliosis and suggestive of ongoing neurodegenerative processes (Figure 3, F and G, and Supplemental Figure 3, D and E). Furthermore, SHMT2 knockdown markedly increased the number of cleaved caspase-3^+^ cells within GFP^+^ AAV-infected regions, reflecting enhanced neuronal apoptosis (Figure 3H and Supplemental Figure 3F). To evaluate motor function, we conducted behavioral tests in SHMT2-knockdown mice. In the rotarod test, mice injected with AAV-shSHMT2 exhibited a substantial reduction in latency to fall, indicating impaired motor coordination and balance (Figure 3I and Supplemental Figure 3G). Similarly, beam-walking performance was compromised, as SHMT2-knockdown mice required more time to cross the beam compared with controls (Figure 3J and Supplemental Figure 3H). In the open-field test, SHMT2-knockdown mice showed reduced total distance traveled, fewer center entries, and decreased center distance, reflecting impairments in locomotion and exploratory behavior (Figure 3, K–M, and Supplemental Figure 3, I–K). Collectively, these findings reveal that SHMT2 knockdown in vivo impairs MSN integrity, induces reactive gliosis and neuronal apoptosis, and ultimately results in motor dysfunction.

### SHMT2 overexpression ameliorates neurodegeneration both in vivo and in vitro.

**Figure 4:**
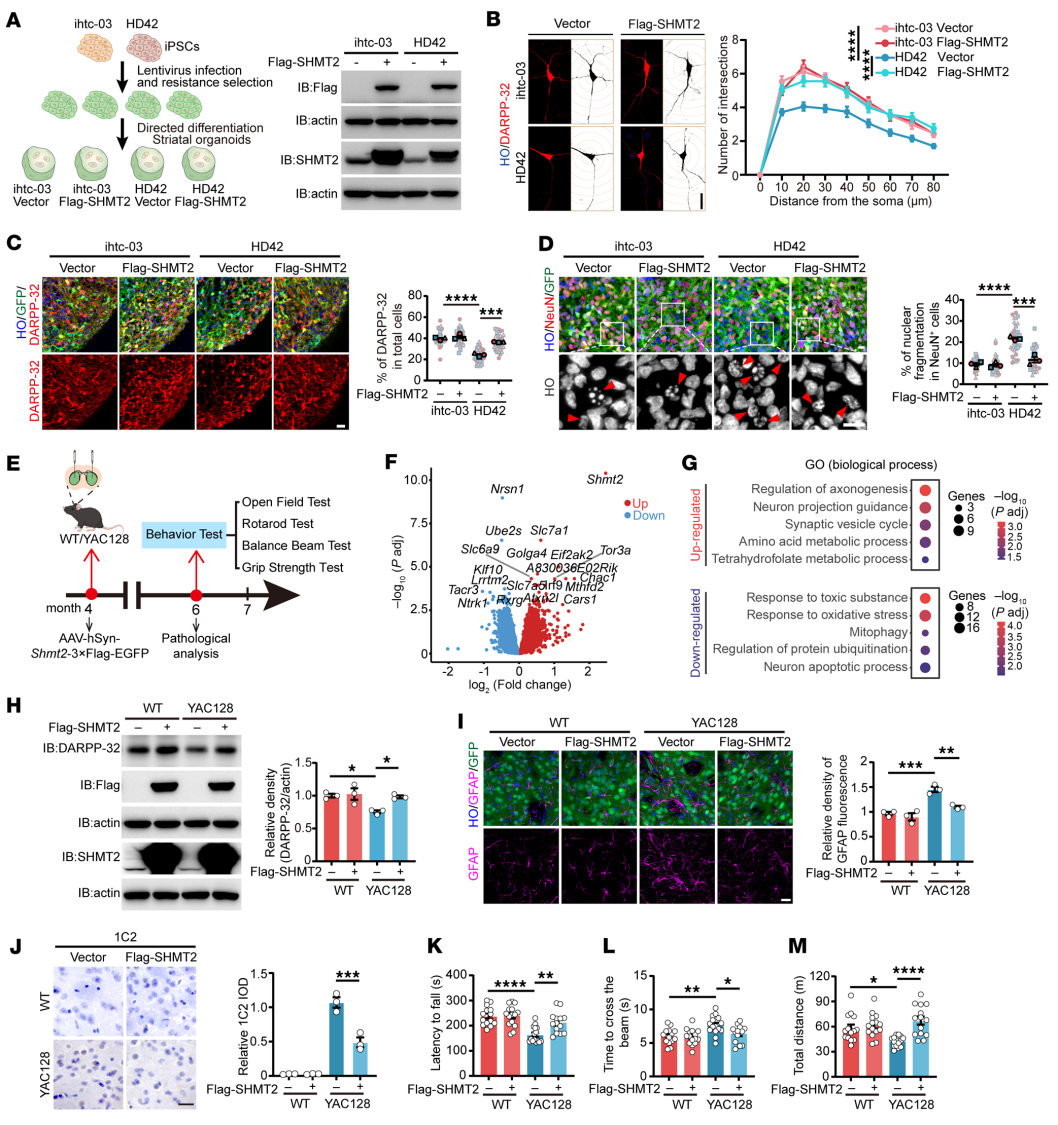
SHMT2 overexpression ameliorates neurodegeneration both in vivo and in vitro. (**A**) Schematic of lentiviral SHMT2 overexpression in control and HD iPSCs and subsequent hSO differentiation; SHMT2 protein confirmed by Western blot. (**B**) Neuronal complexity in control hSOs (con-hSOs) and HD-hSOs with or without SHMT2 overexpression, quantified by Sholl analysis (scale bar: 20 μm; *n* = 60). (**C**) DARPP-32 and GFP costaining in con-hSOs and HD-hSOs with or without SHMT2 overexpression at D60 (scale bar: 20 μm; *n* = 3). (**D**) Nuclear fragmentation in control and SHMT2-overexpressing con-hSOs and HD-hSOs at D60 (scale bar: 10 μm; *n* = 3). (**E**) Timeline of AAV-Con and AAV-SHMT2 stereotaxic injections into the striatum of 4-month-old WT and YAC128 mice. (**F**) Volcano plot illustrating differential gene expression in striatal tissue from YAC128 mice with or without SHMT2 overexpression. DEG cutoff: |log_2_FC| ≥ 0.25, *P*_adj_ ≤ 0.05. (**G**) GO Biological Process enrichment of DEGs from YAC128 mice with or without SHMT2 overexpression. (**H**) DARPP-32 protein in the striatum of WT and YAC128 mice following AAV-Con or AAV-SHMT2 injection (*n* = 3). (**I**) GFAP and GFP staining in the striatum of WT and YAC128 mice after AAV-SHMT2 overexpression (*n* = 3; scale bar: 20 μm). (**J**) mHTT immunoreactivity in striatal sections from WT and YAC128 mice with or without AAV-SHMT2 overexpression (*n* = 3; scale bar: 20 μm). (**K**–**M**) Behavioral assessments 2 months after AAV-Con or AAV-SHMT2 injection (*n* = 14–15). Tests included (**K**) rotarod test, (**L**) balance beam test, and (**M**) open-field test. Data are shown as the mean ± SEM. Two-way ANOVA with Šídák’s test was used for **B**; 1-way ANOVA with Tukey’s test was used for **C**, **D**, and **H**–**M**. *P < 0.05, **P < 0.01, ***P < 0.001, ****P < 0.0001.

To explore the effect of SHMT2 on HD-related pathology, we first introduced lentivirus-mediated SHMT2 overexpression into control and HD patient–derived iPSCs, followed by the selection of SHMT2-expressing cells and subsequent differentiation into hSOs (Figure 4A). In HD-hSOs, we observed reduced neurite outgrowth and structural abnormalities, indicative of compromised neuronal integrity (Figure 4B). Notably, SHMT2 overexpression enhanced neurite length and branching, suggesting a protective role in maintaining neuronal structure and stability (Figure 4B and Supplemental Figure 4A). Immunostaining analyses revealed that SHMT2 overexpression increased the proportion of DARPP-32^+^ and CTIP2^+^ neurons in HD-hSOs (Figure 4C and Supplemental Figure 4, B–E). In parallel, it markedly reduced the number of cells exhibiting nuclear fragmentation (Figure 4D and Supplemental Figure 4, F and G), suggesting that SHMT2 promotes neuronal identity and mitigates HD-associated cellular degeneration. To determine whether these cellular improvements translate into functional benefits in vivo, we next assessed both molecular and behavioral outcomes in YAC128 HD mice following AAV-SHMT2 delivery (Figure 4E). Transcriptomic profiling revealed that SHMT2 overexpression partially reversed YAC128-associated defects in pathways related to dendritic development, synapse organization, and mitochondrial stress responses (Figure 4, F and G, Supplemental Figure 4H, and Supplemental Table 5). Western blot analysis showed that DARPP-32 levels in the striatum were restored upon SHMT2 overexpression, indicating improved MSN integrity (Figure 4H). SHMT2 overexpression also attenuated astrocytic reactivity and reduced mutant huntingtin (mHTT) immunoreactivity and insoluble mHTT levels within GFP^+^ striatal regions of YAC128 mice, consistent with a broader amelioration of HD-related pathological features (Figure 4, I and J, and Supplemental Figure 4I). Functionally, YAC128 mice treated with AAV-SHMT2 exhibited markedly improved motor performance. In the rotarod test, mice displayed substantially prolonged latency to fall, reflecting enhanced coordination (Figure 4K). Beam-walking analysis showed shorter crossing times, indicative of improved balance (Figure 4L). Grip strength was also clearly improved compared with control mice (Supplemental Figure 4J). Finally, in the open-field test, treated animals traveled longer total distances, moved more frequently in the center zone, and made more center entries (Figure 4M and Supplemental Figure 4, K and L). Together, these findings identify SHMT2 as a key modulator of neuronal structure, function, and survival in HD, capable of counteracting MSN degeneration and ameliorating behavioral deficits.

### SHMT2 deficiency drives neurodegeneration via HCY accumulation.

**Figure 5:**
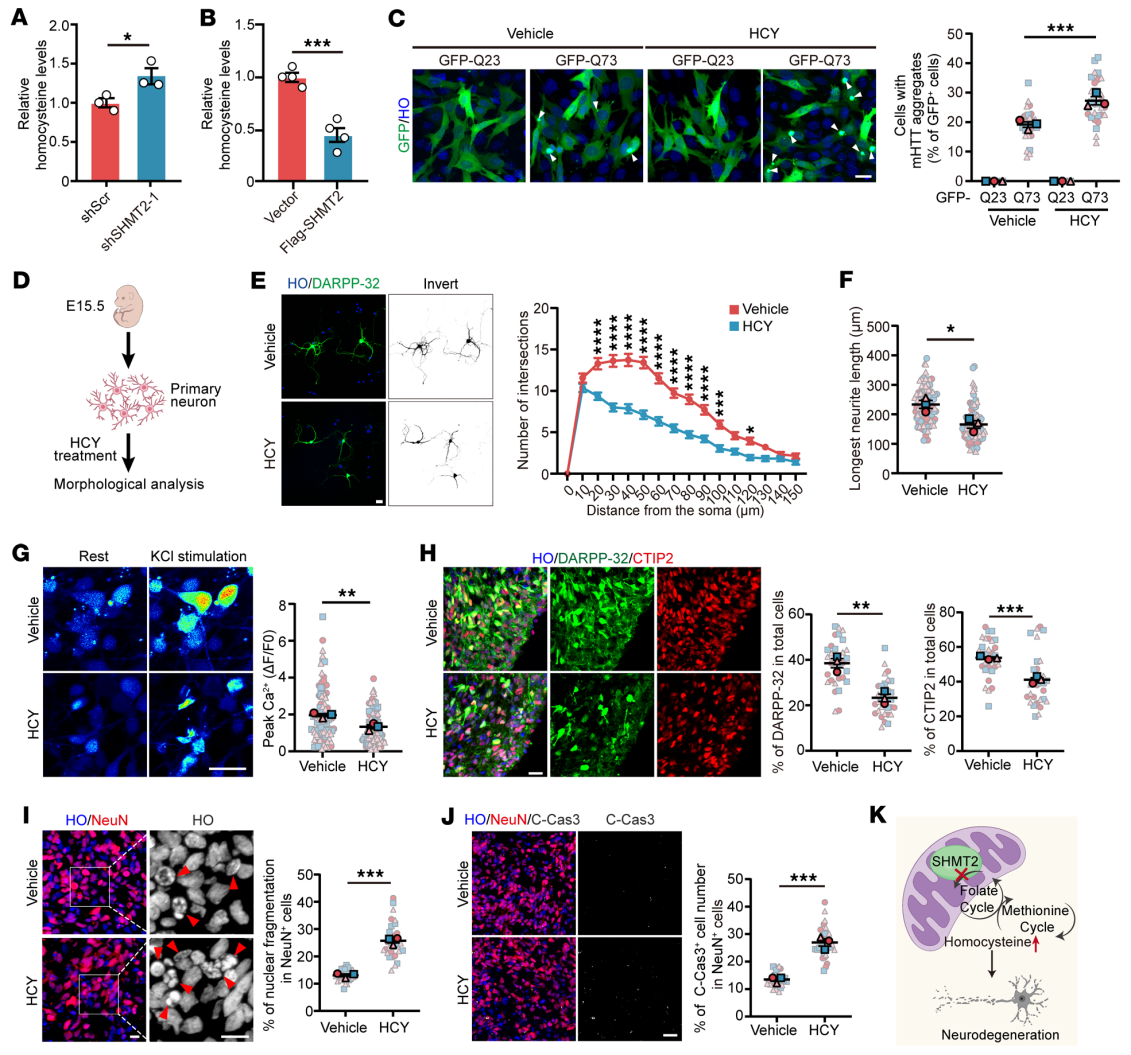
SHMT2 deficiency drives neurodegeneration via HCY accumulation. (**A**) HCY levels were measured in control and SHMT2-knockdown HdhQ7 cells (*n* = 3). (**B**) HdhQ111 cells transfected with Flag-SHMT2 for 48 hours exhibited substantially reduced HCY levels (*n* = 4). (**C**) Representative images and quantification of polyQ aggregates in control and HCY-treated HdhQ7 cells expressing GFP-HTTEx1-Q23 or -Q73. Arrows mark aggregates; data show the percentage of GFP^+^ cells with aggregates (scale bar: 20 μm; *n* = 3). (**D**) Schematic illustration of HCY treatment in primary striatal neurons. (**E** and **F**) Representative images and Sholl analysis of primary striatal neurons treated with 500 μM HCY for 48 hours, with quantification of longest neurite length (scale bar: 20 μm; 3 biological replicates from 60–91 neurons per group). (**G**) Reduced KCl-evoked Ca^2+^ responses in striatal organoid–derived neurons treated with 500 μM HCY for 48 hours compared with controls (scale bar: 10 μm; *n* = 3). (**H**) Immunofluorescence analysis of DARPP-32^+^ (green) and CTIP2^+^ (red) cells in striatal organoids treated with or without HCY (scale bar: 20 μm; *n* = 3). (**I**) Representative images showing nuclear fragmentation in HCY-treated hSOs; arrows mark fragmented nuclei (scale bar: 20 μm; *n* = 3). (**J**) Immunofluorescence and quantification of cleaved caspase-3^+^ cells in control and HCY-treated hSOs (scale bar: 20 μm; *n* = 3). (**K**) Schematic summary of SHMT2 deficiency–induced neurodegeneration. Data are shown as mean ± SEM. Unpaired *t* test was used for **A**, **B**, and **F**–**J**; 1-way ANOVA with Tukey’s test was used for **C**; 2-way ANOVA with Šídák’s test was used for **E**. *P < 0.05, **P < 0.01, ***P < 0.001, ****P < 0.0001.

HCY is a key intermediate metabolite in the 1C metabolic pathway and has been implicated in neurotoxicity and neurodegeneration (22, 28, 29). Targeted metabolomic analysis revealed that HCY levels were substantially elevated in HD models compared with controls (Supplemental Figure 1A). Given that SHMT2 is a central regulator of mt-1C metabolism, we hypothesized that SHMT2 deficiency in HD could disrupt 1C metabolic homeostasis, leading to HCY accumulation. Supporting this hypothesis, we found that SHMT2 knockdown led to an increase in intracellular HCY levels (Figure 5A), whereas SHMT2 overexpression in HdhQ111 cells effectively reduced HCY concentrations (Figure 5B). To examine the effects of HCY on HD-related pathology, we first investigated whether HCY modulates mHTT aggregation. Using a Q73-overexpressing HdhQ7 cell model, we found that HCY treatment markedly increased the number of cells with polyQ aggregates (Figure 5C), suggesting that elevated HCY levels may exacerbate proteotoxic stress in HD. To further assess the effect of HCY on neuronal integrity, we treated primary MSNs with HCY and observed pronounced morphological alterations, including reduced neurite complexity and a substantial shortening of the longest neurite (Figure 5, D–F), indicative of neuronal atrophy and early-stage degeneration. Intracellular calcium imaging revealed that HCY-treated neurons derived from hSOs exhibited a blunted KCl-induced calcium influx, reflecting impaired neuronal excitability and synaptic signaling (Figure 5G). In addition, HCY exposure resulted in a reduction of DARPP-32^+^ and CTIP2^+^ neuronal populations, indicating MSN loss and disruption of striatal neuronal identity (Figure 5H). Furthermore, HCY treatment increased nuclear fragmentation and elevated cleaved caspase-3 levels in NeuN^+^ neurons within hSOs, confirming enhanced apoptotic activity and neuronal cell death (Figure 5, I and J). Collectively, these findings suggest that SHMT2 deficiency leads to HCY accumulation, which exacerbates polyQ aggregation, impairs neuronal structure and function, and promotes neurodegeneration through apoptotic pathways (Figure 5K).

### HCY suppresses histone lactylation through AARS1.

**Figure 6:**
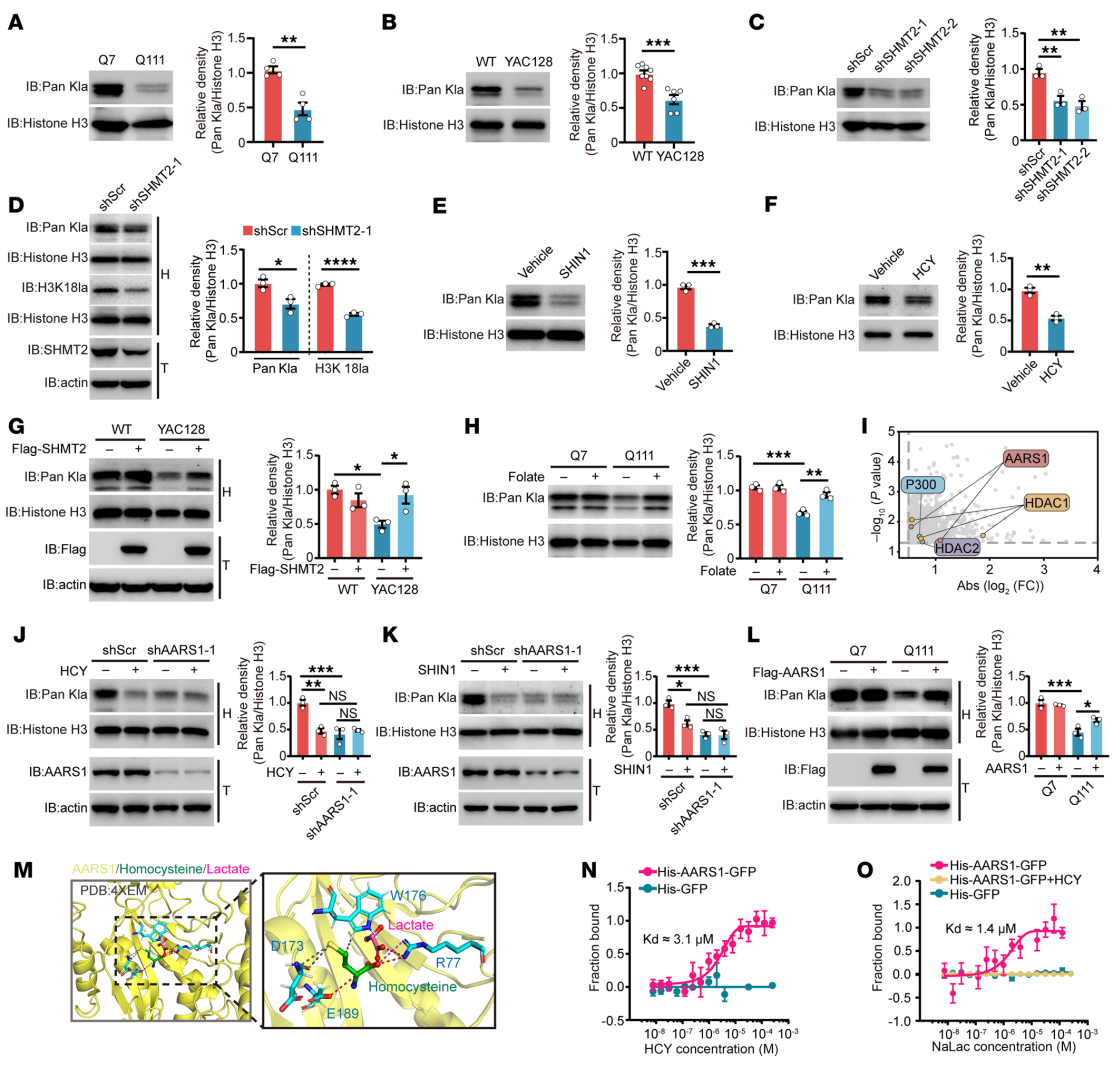
HCY suppresses histone lactylation through AARS1. (**A**) Comparative immunoblot of pan-histone lactylation in HdhQ111 versus HdhQ7 cells (*n* = 4). (**B**) Pan-histone lactylation in striatal tissues from 6-month-old WT and YAC128 mice (*n* = 6–8). (**C**) Pan-histone lactylation in SHMT2-knockdown HdhQ7 cells compared with control (*n* = 3). (**D**) Analysis of pan-histone lactylation and H3K18la in control and SHMT2-knockdown mouse striatum (*n* = 3). (**E**) Pan-histone lactylation in HdhQ7 cells treated with vehicle or SHIN1 (10 μM) for 48 hours (*n* = 3). (**F**) Pan-histone lactylation levels in HdhQ7 cells treated with or without HCY (500 μM) for 48 hours (*n* = 3). (**G**) Pan-histone lactylation in striatal tissues from 6-month-old WT and YAC128 mice injected with AAV-Con or AAV-SHMT2 (*n* = 3). (**H**) Folate supplementation (100 μM, 48 hours) partially restores pan-histone lactylation in HdhQ111 cells (*n* = 3). (**I**) Scatter plot of candidate HCY-binding proteins identified by LiP-MS analysis. (**J**) Histone lactylation in AARS1-knockdown or control HdhQ7 cells treated with or without HCY (500 μM, 48 hours) (*n* = 3). (**K**) Pan-histone lactylation in control and AARS1-knockdown HdhQ7 cells treated with or without SHIN1 (10 μM, 48 hours) (*n* = 3). (**L**) Histone lactylation in HdhQ7 and HdhQ111 cells transfected with Flag-AARS1 or empty vector (*n* = 3). (**M**) Molecular docking showing HCY binding to human AARS1 (PDB: 4XEM) at the predicted lactate binding site. (**N**) Microscale thermophoresis (MST) analysis of AARS1-HCY interaction using gradient HCY; representative MST curves are shown. (**O**) MST analysis of AARS1 with or without HCY in the presence of gradient sodium lactate; representative MST curves shown. Data are shown as the mean ± SEM. Unpaired *t* test was used for **A**, **B**, and **D**–**F**; 1-way ANOVA with Tukey’s test was used for **C**, **G**, **H**, and **J**–**L**. *P < 0.05, **P < 0.01, ***P < 0.001, ****P < 0.0001.

Histone lactylation is an emerging posttranslational modification that connects metabolic states to transcriptional regulation, playing critical roles in neuronal function and homeostasis (30, 31). Metabolic intermediates can modulate histone modifications by affecting the activity of associated enzymes (32–34). In this context, we speculated that elevated HCY levels resulting from impaired 1C metabolism might contribute to the dysregulation of histone lactylation. To test this, we first assessed lactylation levels in HdhQ7 and HdhQ111 striatal cells as well as in the striatum of WT and YAC128 mice. Compared with controls, both HdhQ111 cells and YAC128 mouse striata showed markedly reduced histone lactylation (Figure 6, A and B), indicating consistent epigenetic disruption across HD models. SHMT2 knockdown or pharmacological inhibition using SHIN1 produced similar reductions in histone lactylation (Figure 6, C–E), implicating SHMT2 deficiency as a contributing factor. Notably, site-specific lactylation analysis revealed a broad decline in HdhQ111 cells, closely mirrored by SHIN1-treated cells (Supplemental Figure 5, A and B). HCY treatment further suppressed histone lactylation (Figure 6F), reinforcing the link between HCY accumulation and epigenetic perturbation. Importantly, SHMT2 overexpression restored lactylation levels in HD models (Figure 6G and Supplemental Figure 5, C and D). Complementarily, folate supplementation, by enhancing folate cycle activity and reducing HCY accumulation, also rescued histone lactylation defects in both HD models and SHMT2-deficient conditions (Figure 6H and Supplemental Figure 5E), underscoring the critical role of HCY homeostasis in regulating this epigenetic modification. To further elucidate the mechanisms by which HCY influences histone lactylation levels, we performed limited proteolysis–mass spectrometry (LiP-MS) analysis (Supplemental Figure 5F), which identified 1,309 markedly altered peptides (Supplemental Table 6) corresponding to several potential HCY-interacting proteins, including AARS1, p300, HDAC1, and HDAC2 — all of which have been previously implicated in histone lactylation regulation (Figure 6I). To validate their functional relevance in striatal cells, we individually knocked down each of these candidates. Notably, silencing p300, HDAC1, or HDAC2 had minimal effect on histone lactylation, whereas AARS1 knockdown markedly suppressed global histone lactylation levels, suggesting a specific role for AARS1 in this context (Figure 6J and Supplemental Figure 5, G and H). Furthermore, AARS1 depletion abolished the HCY-induced reduction in histone lactylation (Figure 6J and Supplemental Figure 5I), indicating that AARS1 mediates the epigenetic effects of HCY accumulation. Moreover, AARS1 knockdown prevented the reduction in histone lactylation caused by SHMT2 knockdown or SHIN1 treatment (Figure 6K and Supplemental Figure 5, J and K). In contrast, overexpression of AARS1 in HdhQ111 cells led to a notable increase in histone lactylation levels (Figure 6L). Interestingly, despite these functional effects, AARS1 protein levels remained unchanged across multiple HD models (Supplemental Figure 5, L–N), suggesting that HCY may regulate histone lactylation through modulating AARS1 activity rather than altering its expression. To further explore the mechanism by which HCY modulates AARS1 activity, we performed molecular docking analysis of HCY and lactate with the crystal structure of AARS1. The results showed spatial overlap between HCY and lactate at the R77 residue, suggesting a potential competitive binding mechanism in which HCY may displace lactate at the AARS1 active site to exert its functional effects (Figure 6M). Microscale thermophoresis (MST) analysis revealed that HCY can directly bind to AARS1 and competitively interfere with lactate binding, providing direct evidence that HCY disrupts AARS1-mediated histone lactylation through competitive binding (Figure 6, N and O, and Supplemental Figure 5O). Together, these results demonstrate that elevated HCY competitively disrupts AARS1-mediated histone lactylation, contributing to the epigenetic dysregulation observed in HD models.

### Haloperidol alleviates metabolic-epigenetic dysregulation via SHMT2-dependent mechanisms.

**Figure 7:**
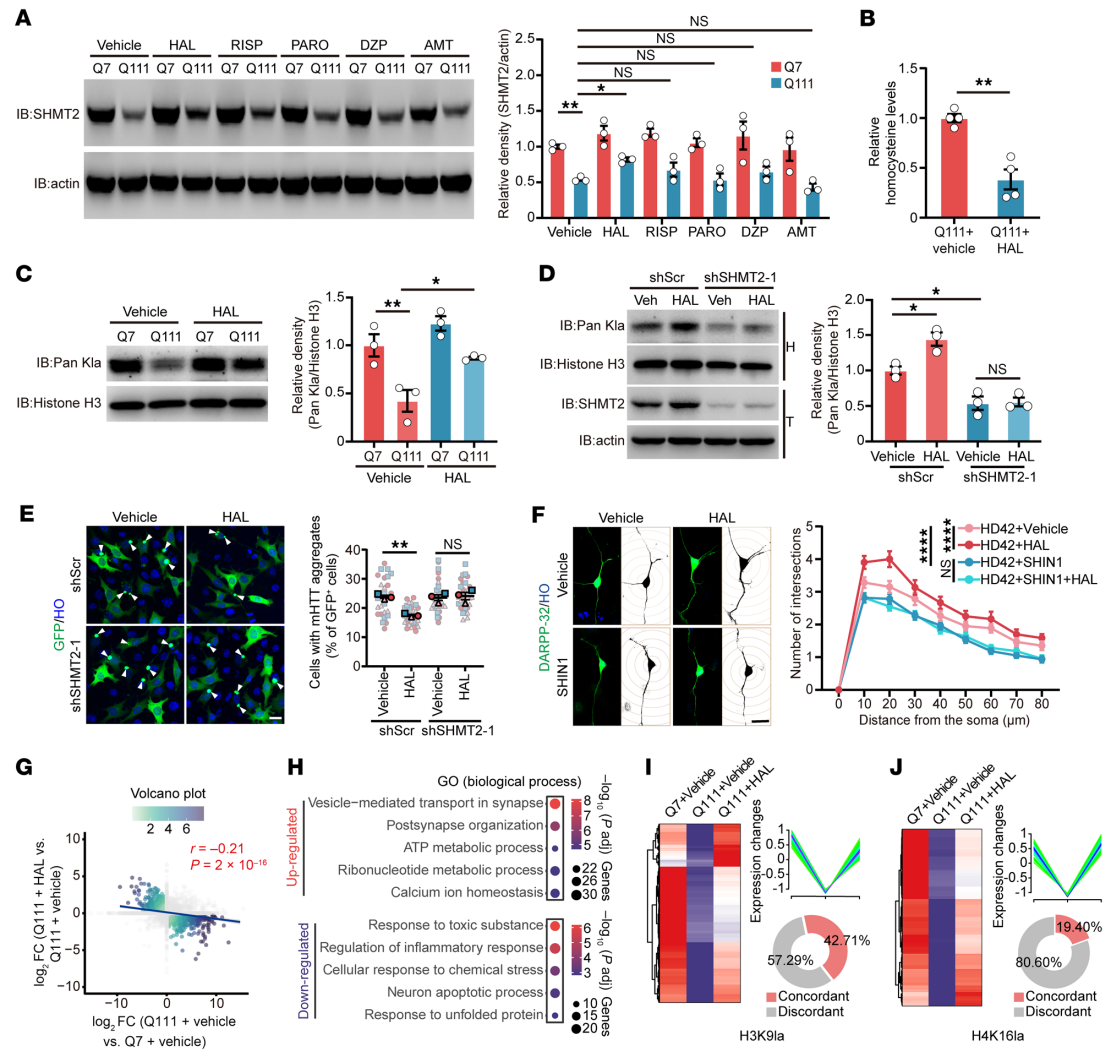
Haloperidol alleviates metabolic-epigenetic dysregulation via SHMT2-dependent mechanisms. (**A**) SHMT2 expression in HdhQ7 and HdhQ111 cells after 48-hour treatment with commonly used HD medications (*n* = 3). HAL, haloperidol; RISP, risperidone; PARO, paroxetine; DZP, donepezil; AMT, amantadine. (**B**) HCY levels in vehicle- and haloperidol-treated (20 μM, 48 h) HdhQ111 cells (*n* = 4). (**C**) Pan-histone lactylation levels in HdhQ7 and HdhQ111 cells treated with haloperidol (20 μM) for 48 hours (*n* = 3). (**D**) Pan-histone lactylation in HdhQ111 cells with or without SHMT2 knockdown following haloperidol treatment (20 μM, 48 hours) (*n* = 3). (**E**) Images of GFP-HTTEx1-Q73–expressing HdhQ111 cells with SHMT2 knockdown followed by haloperidol treatment; polyQ aggregates were quantified (scale bar: 20 μm; *n* = 3). (**F**) Sholl analysis of neurons derived from HD-hSOs treated with or without SHIN1, followed by haloperidol treatment (scale bar: 20 μm; *n* = 60). (**G**) Dual volcano plot comparing gene expression between Q111 + vehicle and Q7 + vehicle as well as Q111 + HAL and Q111 + vehicle. Blue line indicates regression curve with negative correlation (*r* = –0.21). DEG cutoff: |log_2_FC| ≥ 0.25, *P*_adj_ ≤ 0.05. (**H**) GO Biological Process enrichment of negatively correlated genes identified in Supplemental Figure 6H. (**I** and **J**) Integration of CUT&Tag and RNA-seq for H3K9la- and H4K16la-marked genes. Concordant genes were defined as genes identified by RNA-seq as downregulated in Q111 versus Q7 and restored by HAL treatment, with transcriptional changes consistent with histone lactylation–associated chromatin occupancy. Heatmaps and line plots (centroid in blue) depict concordant CUT&Tag and RNA-seq expression changes. Donut plots summarize the proportion of concordant versus discordant genes. Data are shown as the mean ± SEM. One-way ANOVA with Tukey’s test was used for **A**, and **C**–**E**; unpaired *t* test was used for **B**; 2-way ANOVA with Šídák’s test was used for **F**. *P < 0.05, **P < 0.01, ****P < 0.0001.

To determine whether symptomatic medications relevant to HD modulate SHMT2-regulated histone lactylation in vitro, we screened a panel of pharmacological agents, including haloperidol, risperidone, amantadine, paroxetine, and donepezil, which are commonly used for symptomatic management in HD. Among these agents, only haloperidol markedly upregulated SHMT2 expression in HdhQ111 cells, whereas the other drugs exhibited no notable effects (Figure 7A). However, haloperidol treatment did not substantially alter *Shmt2* mRNA levels in HdhQ7 and HdhQ111 cells, indicating that haloperidol may regulate SHMT2 at the protein level (Supplemental Figure 6A). Furthermore, haloperidol treatment reduced intracellular HCY levels in HdhQ111 cells compared with untreated controls and restored histone lactylation levels that were decreased in HdhQ111 cells (Figure 7, B and C). Importantly, inhibition of SHMT2 by genetic knockdown abolished the haloperidol-induced increase in histone lactylation (Figure 7D), suggesting that haloperidol alleviates metabolic-epigenetic dysregulation in HD models by modulating the SHMT2-HCY axis. Immunofluorescence staining revealed that haloperidol treatment clearly reduced the number of cells harboring polyQ aggregates, and this effect was abolished by SHMT2 knockdown (Figure 7E and Supplemental Figure 6B). Consistently, pharmacological inhibition of SHMT2 with SHIN1 also blocked the haloperidol-mediated restoration of neuronal morphology (Figure 7F and Supplemental Figure 6, C–E), indicating that these effects require SHMT2 activity. Given that haloperidol restores histone lactylation, which is closely linked to transcriptional regulation, we next examined whether its effects are mediated through histone-lactylation–dependent changes in gene transcription. To address this, we treated HdhQ111 cells with haloperidol and performed RNA-seq on Q7, Q111, and Q111 plus haloperidol groups (Supplemental Figure 6F and Supplemental Table 7). A dual volcano plot comparing Q111 versus Q7 and Q111+HAL versus Q111 revealed a set of genes showing reversed expression patterns in response to haloperidol, with a negative correlation (*r* = –0.21) between the 2 comparisons, supporting its partial restoration of transcriptional homeostasis (Figure 7G). Heatmap visualization of the overlapping genes further supported this observation, showing that many of the affected genes were downregulated in Q111 relative to Q7 and subsequently upregulated upon haloperidol treatment, or vice versa—indicative of gene-level rescue effects (Supplemental Figure 6G). Of the 4,012 differentially expressed genes (DEGs) identified in the Q111+HAL versus Q111 comparison, 1,312 overlapped with DEGs from the Q111 versus Q7 group and exhibited inverse expression trends. These genes were defined as haloperidol-rescued targets and accounted for approximately 33% of the HAL-regulated transcriptome (Supplemental Figure 6H). Gene ontology analysis of the up- and downregulated gene sets identified pathways related to synaptic organization, metabolic processes, calcium ion homeostasis, inflammatory and stress responses, and neuronal apoptosis (Figure 7H). Combined cleavage under targets and tagmentation assay–RNA-seq (CUT&Tag–RNA-seq) analysis showed that a substantial proportion of genes marked by H3K9la or H4K16la exhibited concordant expression changes upon haloperidol treatment, indicating that haloperidol-induced transcriptional responses partially align with its effects on histone lactylation (Figure 7, I and J, and Supplemental Table 8). Gene Ontology and pathway enrichment analyses of these concordant genes further revealed enrichment in functional categories related to neuronal organization, synaptic and vesicle-associated processes, and cellular metabolic regulation (Supplemental Figure 6, I and J). Together, these findings suggest that haloperidol partially restores SHMT2-dependent metabolic and epigenetic homeostasis in HD models.

## Discussion

1C metabolism is a fundamental biochemical network that integrates nutrient availability with epigenetic regulation (35). It encompasses the folate and methionine cycles and generates SAM, the universal methyl donor required for DNA, histone, and RNA methylation (36–38). Through these modifications, 1C metabolism plays a critical role in gene regulation, chromatin architecture, and cellular homeostasis (39–42). SHMT2 is a mitochondrial enzyme essential for serine-to-glycine conversion and 1C unit production (14, 15, 43), and its mutations have been linked to developmental disorders affecting the brain and heart (17, 44). However, its role in neurodegenerative conditions has remained elusive. Our findings delineate a previously unrecognized metabolic and epigenetic axis in HD, in which mt-1C metabolism, mediated by SHMT2, governs histone lactylation through modulation of HCY and AARS1 activity. This epigenetic disruption contributes to neuronal vulnerability and pathological polyQ aggregation. Notably, the clinically used antipsychotic haloperidol partially restores this axis by upregulating SHMT2, reducing HCY accumulation, and reinstating histone lactylation, which is associated with improved neuronal morphology. These results expand the functional landscape of SHMT2 beyond its traditional metabolic role and highlight its central position in linking mitochondrial function with chromatin regulation during neurodegeneration.

Histone lactylation has emerged as a crucial posttranslational modification that links cellular metabolism to gene regulation, particularly in neurological disorders (45). In AD, histone H4K12 lactylation modulates microglial glucose metabolism and inflammatory responses (46). Additionally, lactylation at APP-K612 influences amyloid processing by disrupting its interaction with BACE1 and enhancing lysosomal degradation, which ultimately reduces Aβ pathology and improves cognitive outcomes (47). Tau-K677 lactylation has also been shown to regulate ferritinophagy and ferroptosis via the MAPK signaling pathway. Mutations at this site alleviate neuronal injury and cognitive decline, highlighting its potential therapeutic relevance (48). Beyond AD, lactylation contributes to neuroprotection in ischemic stroke, where astrocytic LRP1 suppresses lactate production and ARF1 lactylation, thereby promoting mitochondrial transfer to neurons (49). Our data provide additional evidence for dysregulated histone lactylation in HD, supporting a mechanistic link between altered epigenetic regulation and disease progression. This imbalance may contribute to widespread transcriptional disruption, metabolic dysfunction, and increased neuronal susceptibility, underscoring the need to further investigate the role of lactylation in chromatin remodeling and neurodegeneration.

AARS1, a core enzyme in aminoacyl-tRNA synthesis, is essential for accurate protein translation, cellular stress responses, and homeostasis (50). While best known for its canonical role in translation fidelity, AARS1 dysfunction has been implicated in a broad range of conditions, including Charcot-Marie-Tooth disease, cancer, and metabolic syndromes involving the mTOR pathway (51–53). Recent studies have uncovered an additional function of AARS1 in regulating histone lactylation, particularly through modulation of p53 activity and metabolic signaling (54). Our findings demonstrate that HCY competes with lactate for AARS1 binding, thereby interfering with AARS1-mediated lactylation of histones (55). These results suggest the existence of a novel metabolic-epigenetic interaction mediated by AARS1. However, whether HCY alters the aminoacylation activity of AARS1 remains to be determined and warrants further investigation.

The observation that haloperidol restores SHMT2 expression and histone lactylation highlights the potential to pharmacologically modulate metabolic and epigenetic pathways in HD models. Whether other agents, such as folate-cycle modulators, SAM analogs, or AARS1-specific regulators, can replicate or enhance these effects remains to be tested. Given that histone lactylation is implicated in multiple neurological diseases, lactylation signatures may also hold promise as epigenetic biomarkers for disease staging or treatment response monitoring. Together, these insights expand our understanding of how mitochondrial metabolism interfaces with chromatin regulation and open avenues for developing mechanism-based therapeutic strategies for HD and potentially other neurodegenerative disorders.

### Limitations of the study.

Our work establishes a mechanistic link between SHMT2-dependent 1C metabolism and histone lactylation via AARS1, yet several questions remain. First, although SHMT2 downregulation was reproducibly observed across HD models, the mechanism by which mHTT suppresses SHMT2 remains unknown. Transcriptional repression is plausible, but effects on mRNA stability or protein turnover cannot be excluded. Second, although our models span CAG repeat lengths from 42 to 128, we did not determine whether the magnitude of SHMT2 deficiency or downstream metabolic-epigenetic alterations scales with repeat length, which will require systematic and controlled analyses across a broader CAG range. Third, it remains unclear how SHMT2 deficiency intersects with somatic CAG expansion, a major driver of HD pathogenesis. SHMT2 loss could influence DNA repair pathways that mediate repeat instability, or conversely, expansion-induced cellular stress may impair SHMT2 expression. In addition, the Q111/Q7 striatal cell model used for metabolomic analyses has inherent limitations in recapitulating the full spectrum of in vivo molecular signatures, particularly the complex transcriptional, metabolic, and cell-type–specific alterations observed in HD mouse brains and human tissues. To partially address these limitations, we incorporated transcriptomic data from patient iPSC–derived striatal organoids, which more closely resemble human MSNs in both cellular identity and functional pathways. The organoid dataset provides independent validation of key pathway-level perturbations and helps bridge the gap between simplified cellular systems and more physiologically relevant HD models.

## Methods

### Sex as a biological variable.

In this study, sex was not considered as a biological variable. Only male mice were used to minimize estrous cycle–related hormonal variability and enhance experimental reproducibility.

### Reagents and antibodies.

SHIN1 (HY-112066) was purchased from MedChemExpress. HCY (69453), folate (F8758), haloperidol (H1512), risperidone (R3030), paroxetine (P9623), donepezil (D6821), and amantadine (A1260) were obtained from Sigma-Aldrich. The protease inhibitor cocktail (P001) was obtained from NCM Biotech. GenEscort I transfection reagent (WIS1600) was purchased from Wisegen. A full list of primary and secondary antibodies used in this study is provided in Supplemental Table 9.

### Cell culture.

HEK293 cells were obtained from the ATCC and cultured in DMEM supplemented with 10% FBS and 1% penicillin/streptomycin at 37°C in a humidified atmosphere with 5% CO_2_. Primary striatal neurons were isolated from embryonic day 15.5 to 16.5 (E15.5–E16.5) mouse embryos. Neurons were plated onto 24-well glass coverslips precoated with poly-D-lysine (100 μg/mL) and laminin (15 μg/mL) and maintained in Neurobasal medium supplemented with 2% B27 and 0.5 mM GlutaMAX. Human pluripotent stem cell (hPSC) lines, including control lines (ihtc-03, IMR90-4, and RC01001-A), and HD patient–derived iPSC lines (HD40, HD42, and HD66), were used in this study (Supplemental Table 10). hPSCs were cultured under feeder-free conditions on vitronectin-coated plates (Thermo Fisher Scientific) in Essential 8 (E8) medium (Life Technologies), with daily half-volume medium changes. Colonies were passaged every 5–7 days using EDTA (STEMCELL Technologies). Mouse striatal precursor cell lines HdhQ7 and HdhQ111 were obtained from the Coriell Institute. These cells were maintained at 33°C in DMEM supplemented with 10% FBS, 1% penicillin/streptomycin, and 400 μg/mL Geneticin (G418, Yeasen) in a humidified 5% CO_2_ atmosphere.

### Plasmids and transfection.

Full-length *SHMT2* and *AARS1* cDNAs were amplified by PCR and subcloned into the pcDNA3.1(+)-Flag vector. Full-length *AARS1* was also cloned into the pET-28a(+) vector with an N-terminal 6×His tag and a C-terminal EGFP-6×His tag. The constructs HTTEx1-Q23-Myc (HTT-Q23) and HTTEx1-Q73-Myc (HTT-Q73) were obtained from the Coriell Institute for Medical Research. GFP-tagged versions of HTTEx1-Q23 and HTTEx1-Q73 were generated by replacing the Myc tag with a GFP tag in the original constructs. For plasmid transfection, DNA was diluted in serum-free DMEM, followed by the addition of GenEscort I transfection reagent (WIS1600) at a ratio of 2 μL reagent per 1 μg DNA. The mixture was gently mixed and incubated at room temperature for 15 minutes before being added to the cells. Cells were harvested 48 or 72 hours after transfection for downstream assays.

### Bulk RNA-Seq.

Cell pellets (≥1 × 10^6^ cells per group) were collected, and total RNA was extracted using TRIzol reagent (Sigma-Aldrich) according to the manufacturer’s instructions. Poly(A)^+^ mRNA was isolated and fragmented under elevated temperature. First- and second-strand cDNA synthesis was performed using reverse transcriptase, followed by end repair, A-tailing, and adaptor ligation with the NEBNext Ultra II RNA Library Prep Kit for Illumina (New England Biolabs). The resulting libraries were PCR-amplified, purified, and subjected to quality control prior to sequencing. Paired-end sequencing (150 bp) was performed on the Illumina NovaSeq X Plus platform (Illumina).

### Metabolomics.

Cell pellets (≥1 × 10^7^ cells per group) were extracted with prechilled methanol/acetonitrile/water (2:2:1, v/v). Samples were sonicated at low temperature for 30 minutes, incubated at –20°C for 10 minutes, and centrifuged at 14,000*g* for 20 minutes at 4°C. The resulting supernatants were collected and vacuum-dried. Prior to mass spectrometry analysis, dried metabolites were reconstituted in 100 μL acetonitrile/water (1:1, v/v), vortexed, and centrifuged again at 14,000*g* for 15 minutes at 4°C. Metabolite separation was performed on an Agilent 1290 Infinity LC ultra-high-performance liquid chromatography system equipped with HILIC and C18 columns. Mass spectrometric detection was conducted using an AB SCIEX 6500+ QTRAP mass spectrometer. Raw data were processed using MultiQuant or Analyst software for peak extraction. Metabolite concentrations were quantified based on the ratio of analyte peak areas to internal standards and calculated using external calibration curves.

### Total protein extraction and Western blot.

Cells or tissues were lysed on ice using RIPA lysis buffer containing 50 mM Tris-HCl (pH 7.5), 150 mM NaCl, and 1% Triton X-100, supplemented with a protease inhibitor cocktail (1:100 dilution). After 30 minutes of lysis, the samples were centrifuged at 13,000 × *g* for 10 minutes at 4°C. The supernatants were collected, and protein concentrations were determined using a BCA protein assay kit (Beyotime) and measured with a microplate reader. Protein samples were normalized to equal concentrations, mixed with SDS loading buffer, and denatured by boiling at 100°C for 10 minutes. After brief centrifugation, equal amounts of protein were loaded onto SDS-PAGE gels for electrophoretic separation. Proteins were then transferred to nitrocellulose membranes and probed with specific primary antibodies against the target proteins.

### CRISPRi knockdown and cell line generation.

A dual-vector CRISPRi system was used to generate the CRISPRi-based ihtc-03 SHMT2 knockdown human iPSC line. The dCas9-KRAB repressor was expressed from a lentiviral vector derived from lentiCRISPR (Addgene #61425), while sgRNAs targeting the region from –50 to +300 bp relative to the *SHMT2* transcription start site were cloned into a separate hU6-sgRNA-SV40-EGFP vector (GV371, GeneChem). The sgRNA sequences used were as follows: SHMT2 knockdown 1 (GGGCGGCTCGGGTAAGAATG), SHMT2 knockdown 2 (TCGCGCATGCGTTCTCCGAA), and a nontargeting control (TTCTCCGAACGTGTCACGT). For the experiments, human iPSCs were first transduced with the dCas9-KRAB lentivirus for 48 hours. One week after transduction, selection was initiated with 2 μg/mL blasticidin S, followed by maintenance in 0.5 μg/mL blasticidin S for 2 weeks to establish stable pools. These dCas9-expressing cells were then transduced with lentiviruses encoding either target or control sgRNAs. After 48 hours of transduction and 2 weeks of culture in E8 medium, cells were dissociated with Accutase to generate a single-cell suspension. GFP^+^ cells were isolated using a BD FACS Aria Fusion instrument after filtration through a cell strainer. Sorted cells were plated on vitronectin-coated plates and cultured in E8 medium supplemented with ROCK inhibitor (STEMCELL Technologies) for subsequent experiments.

### Histone extraction.

Histones were extracted from cells and tissues using an acid extraction protocol as previously described (56). Briefly, samples were lysed in ice-cold lysis buffer containing 10 mM Tris-HCl (pH 8.0), 1 mM KCl, 1.5 mM MgCl_2_, and 1 mM DTT to isolate the nuclear fraction. The isolated nuclei were then incubated with 0.2 M H_2_SO_4_ at 4°C overnight to solubilize histones. Following centrifugation at 16,000*g* for 10 minutes at 4°C, the supernatant was collected, and histones were precipitated by adding ice-cold trichloroacetic acid to a final concentration of 35%. The resulting pellet was washed, air-dried, and resuspended in distilled water for subsequent immunoblotting analysis.

### Quantitative real-time PCR.

Total RNA was extracted using a commercial RNA isolation kit (Ultrapure RNA Kit, CWBIO) following the manufacturer’s instructions. cDNA was synthesized from 0.5 μg total RNA using the HiScript III RT SuperMix (Vazyme) and subsequently diluted 10-fold. Quantitative PCR was carried out using SYBR Green chemistry on the QuantStudio 3 Real-Time PCR System (Applied Biosystems, Thermo Fisher Scientific). Primer sequences used for amplification are provided in Supplemental Table 11.

### mHTT aggregation detection.

To assess mHTT aggregation, cells were seeded onto glass coverslips placed in 12-well plates precoated overnight at 4°C. When cell confluency reached approximately 60%, cells were transfected with HTTEx1-Q23-GFP or HTTEx1-Q73-GFP expression plasmids. After 48 hours, cells were fixed with 4% paraformaldehyde (PFA) for 20 minutes at room temperature, permeabilized with 0.1% Triton X-100 for 5 minutes, and blocked with 1% BSA containing 0.05% Triton X-100 for 1 hour. Cells were then incubated with primary antibodies overnight at 4°C, followed by fluorophore-conjugated secondary antibodies for 1 hour at room temperature. After PBS washes, coverslips were mounted and imaged using fluorescence microscopy. The percentage of cells containing visible aggregates was quantified using ImageJ software. For immunoblotting analysis of mHTT aggregation, cells were harvested and lysed in RIPA buffer. Following centrifugation, the supernatant was collected as the soluble protein fraction. The pellet containing insoluble aggregates was resuspended and solubilized in buffer containing 8 M urea, 4% SDS, 0.125 M Tris-HCl (pH 6.8), 12 mM EDTA, and 3% β-mercaptoethanol. Samples were mixed with SDS loading buffer, resolved by SDS-PAGE, and analyzed by immunoblotting.

### shRNAs and viral packaging.

The pLKO.1-puro control vector (SHC001) was obtained from Sigma-Aldrich. shRNA constructs targeting *Shmt2* and *Aars1* were generated by inserting the corresponding target sequences (listed in Supplemental Table 11) into the pLKO.1-puro backbone. Lentiviral packaging plasmids pMDLg/pRRE, pRSV-Rev, and pCMV-VSVG were obtained from Addgene. For lentivirus production, HEK293 cells were transfected using GenEscort I with a DNA mixture containing 5 μg shRNA plasmid, 2.5 μg pMDLg/pRRE, 1.25 μg pRSV-Rev, and 1.5 μg pCMV-VSVG. The culture medium was replaced 24 hours after transfection, and viral supernatants were collected after 48 hours. Supernatants were filtered through a 0.45 μm membrane and concentrated by adding 5× PEG8000 at 4°C overnight. The mixture was centrifuged at 17,000 × *g* for 30 minutes at 4°C, and the resulting viral pellet was resuspended in ice-cold PBS, aliquoted, and stored at –80°C for long-term use. For transduction, target cells were infected with the viral supernatant in the presence of polybrene (10 μg/mL). After 48 hours, puromycin was added to select for stable knockdown cell lines. Selected cells were maintained under puromycin selection for at least 48 hours prior to cryopreservation and downstream assays.

### Generation of striatal organoids.

To generate striatal organoids, hPSCs were dissociated using dispase and gently washed with DMEM/F12. The cells were transferred to nonadherent flasks and cultured in a 1:1 mixture of E8 medium and neural induction medium (NIM), consisting of DMEM/F12 supplemented with 1% N2 and 1% non-essential amino acids (Life Technologies), to promote embryoid body (EB) formation. For neural induction, NIM was further supplemented with the SMAD pathway inhibitor SB431542 and the BMP receptor inhibitor DMH1, and half of the medium was replaced daily. On day 7, EBs were transferred to 6-well plates and allowed to attach in NIM supplemented with 10% FBS (Life Technologies). From this point onward, half-volume medium changes were performed every other day using serum-free NIM. By approximately day 10, neural tube–like rosettes were visible under a light microscope. On day 16, rosettes were manually isolated and transferred to suspension culture in NIM containing 2% B27. For striatal induction, Sonic Hedgehog (SHH; R&D Systems) was added at a concentration of 20–200 ng/mL. To obtain single neurons for downstream analysis, mature striatal organoids were dissociated into single-cell suspensions using TrypLE (Life Technologies) and seeded onto Matrigel-coated glass coverslips in 24-well plates at a density of 3 × 10^4^ cells per well. Cells were cultured under standard conditions and subsequently subjected to immunostaining and morphological analysis.

### Calcium imaging.

Neurons derived from striatal organoids differentiated for 6 weeks, following 5 days in vitro (DIV5) of culture, were seeded onto Matrigel-coated confocal imaging dishes (Corning) and incubated with 1 μM Fluo-4 AM (Life Technologies) at 37°C for 15 minutes, followed by an additional 15 minutes at room temperature. After dye loading, cells were gently washed with DPBS (Life Technologies) and transferred to live-cell imaging solution. Time-lapse calcium imaging was performed using a Zeiss LSM 800 confocal fluorescence microscope. Neuronal activation was induced by the addition of 20 μL of 67 mM KCl solution, and time-series images were acquired every 3.2 seconds. Fluorescence intensity over time was quantified using ImageJ (NIH) and GraphPad Prism 8.0.1, and the calcium signaling revealed by the peak Ca^2+^ (Fmax − F0)/F0.

### CUT&Tag.

CUT&Tag was performed using the NovoNGS CUT&Tag 4.0 High-Sensitivity Kit (N259-YH01, Novoprotein) according to the manufacturer’s instructions. Briefly, cells were immobilized with ConA beads and incubated sequentially with primary antibodies against H3K9la or H4K16la and secondary antibodies. ChiTag pAG transposase was then added to fragment the DNA and introduce sequencing adapters. The library was subsequently amplified via PCR with specific index primers. The final library was quality controlled using a Bioanalyzer and sequenced on an Illumina NovaSeq X Plus.

### DAB staining.

In brief, after incubation with a peroxidase (HRP) detection system (DAB Substrate Kit, Vector Laboratories) and rinsing with PBS, the sections were blocked with donkey serum to reduce nonspecific binding. Subsequently, the sections were incubated with the primary antibody overnight at 4°C, followed by incubation with the HRP-conjugated secondary antibody for 1 hour at room temperature. Finally, the sections were incubated with the DAB staining working solution at room temperature for 2–10 minutes, and the reaction was terminated with distilled water. For nuclear counterstaining, sections were immersed in hematoxylin for 5 minutes, followed by rinsing in tap water for bluing. Finally, sections were dehydrated through graded alcohols, cleared in xylene, and mounted with permanent mounting medium.

### Animals.

All animals were maintained under a 12-hour-light/dark cycle with ad libitum access to food and water. All experimental procedures were performed in accordance with institutional guidelines and were approved by the Institutional Animal Care and Use Committee (IACUC2404086) of Nanjing Medical University. Male YAC128 transgenic mice (B6.FVB-Tg [YAC128] 53Hay) were obtained from The Jackson Laboratory.

### Stereotaxic brain injection.

Mice were anesthetized with isoflurane (RWD Life Science) and secured in a stereotaxic frame connected to an anesthesia system. After a longitudinal scalp incision, the skull was leveled using bregma and lambda as reference points. Viral injections were administered bilaterally into the striatum using the following coordinates: anterior-posterior, +0.5 mm; mediolateral, ±2.0 mm; dorsoventral, –3.5 mm. Injections were performed at a rate of 100 nL/min using an automated microinjection system. Following injection, the needle was left in place for 30 minutes to minimize backflow and then slowly withdrawn. The incision was sutured, and mice were placed on a heating pad until full recovery before being returned to their home cages.

### Behavioral experiments.

Behavioral assessments were conducted 1–3 months after stereotaxic viral injection in a dedicated behavioral testing room under quiet and controlled conditions. Mice were housed in groups of no more than 5 with ad libitum access to food and water and acclimated to the testing environment for 2 hours prior to experiments. All tests were performed by an investigator blinded to group identity. Locomotor activity and exploratory behavior were evaluated using the open-field test. Mice were individually placed in a 45 × 45 × 35 cm arena, and their movement was tracked for 10 minutes using ActiTrack V2.7 software (Panlab). Total distance traveled and time spent in the center zone were recorded during a 30-minute session. Motor coordination and balance were assessed using the rotarod test. Mice underwent 2 days of training at a fixed speed (5 rpm), followed by a test day in which the rod accelerated from 5 to 40 rpm over 5 minutes. Latency to fall was recorded across 3 trials, with a maximum cutoff time of 300 seconds. The balance beam test involved a 1-meter-long, 5-mm-wide beam elevated 50 cm above the floor, guiding mice from a brightly lit start platform to a dark goal box containing food. Mice were trained for 2 days, and crossing time was recorded on the test day and averaged across 3 trials. Forelimb grip strength was measured using a grip strength meter. Mice were allowed to grasp a wire grid, and maximal force was recorded as they released the grid. Each mouse underwent 5 consecutive trials; the highest and lowest values were excluded, and the mean of the remaining 3 trials was used for analysis.

### Immunohistochemistry.

Striatal organoids were fixed in 4% PFA at 4°C for 2–4 hours, followed by 3 washes with PBS. Organoids were dehydrated sequentially in 20% sucrose overnight and 30% sucrose until fully submerged at 4°C, and then embedded in OCT compound. Cryosections (10 μm) were prepared and rinsed with PBS to remove residual OCT. Sections were blocked and permeabilized in PBS containing 5% donkey serum and 1% Triton X-100 for 1 hour at room temperature. Primary antibodies diluted in PBS with 5% donkey serum and 0.2% Triton X-100 were applied and incubated overnight at 4°C. After 3 10-minute PBS washes, secondary antibodies and Hoechst 33258 (nuclear counterstain) were added in PBS with 5% donkey serum for 1 hour at room temperature. Sections were washed, mounted, and imaged. For neuronal cultures, cells were fixed in 4% PFA for 20 minutes at room temperature, washed 3 times with PBS (10 minutes each), permeabilized with 0.2% Triton X-100 for 10 minutes, and blocked with 10% donkey serum for 1 hour. Primary antibodies diluted in PBS containing 0.1% Triton X-100 and 5% donkey serum were incubated overnight at 4°C. After PBS washes, secondary antibodies and Hoechst were applied for 1 hour, followed by additional PBS washes and mounting. For brain tissue, mice were anesthetized with tribromoethanol (0.2 mL/10 g body weight) and perfused transcardially with PBS followed by 4% PFA. Brains were post-fixed in 4% PFA overnight at 4°C and cryoprotected in 20% and 30% sucrose for 24–48 hours. Frozen sections were prepared using a cryostat and stored at –20°C. For staining, sections were blocked with 5% donkey serum and 0.3% Triton X-100 for 1 hour, followed by incubation with primary antibodies overnight at 4°C. After PBS washes, secondary antibodies were applied in 5% donkey serum for 1 hour and then washed, mounted, and coverslipped. Fluorescent images were acquired using a Zeiss LSM 800 confocal microscope. Details of primary and secondary antibodies are listed in Supplemental Table 9.

### Measurement of HCY levels.

Total HCY levels were measured using a Total Homocysteine ELISA Kit (Fine Test, Wuhan Fine Biological Technology) according to the manufacturer’s instructions.

### LiP-MS.

HdhQ7 cell pellets (≥2 × 10^6^ cells per group) were collected and lysed. Lysates were centrifuged at 20,000*g* for 10 minutes at 4°C, and supernatants were subjected to protein quantification using the BCA assay. For each group, 100 μg of total protein was incubated with 10 μM HCY at room temperature for 10 minutes. Proteinase K was then added at an enzyme-to-substrate ratio of 1:100 and incubated for 5 minutes at room temperature to perform limited proteolysis. Following proteolysis, samples were treated with 40 mM tris(2-carboxyethyl)phosphine and 100 mM iodoacetamide and then boiled for 5 minutes. Hydroxypropyl-β-cyclodextrin (250 mM), 100 μL of 100 mM ammonium bicarbonate, and trypsin (enzyme-to-substrate ratio 1:50) were added for overnight digestion at 37°C. The reaction was terminated by adjusting the pH below 2 with trifluoroacetic acid. Peptides were separated by Easy-nLC chromatography and analyzed using a Q Exactive HF-X mass spectrometer.

### Protein purification.

His-GFP and His-AARS1-GFP plasmids were transformed into *E*. *coli* strain BL21. Single colonies were picked and cultured in 1.5 mL of LB medium at 37°C for 6–8 hours, then diluted 1:100 into fresh LB broth and incubated overnight at 37°C with shaking at 200 rpm. Protein expression was induced with 0.4 mM isopropyl-β-D-thiogalactopyranoside, followed by overnight incubation at 16°C to promote AARS1 expression. For His-tagged protein purification, bacterial pellets were resuspended in PBST buffer (PBS containing 1% Triton X-100) and lysed by sonication. Lysates were centrifuged at 13,000 × *g* for 15 minutes at 4°C, and the resulting supernatants were applied to HisTrap HP His tag protein purification columns (Cytiva). Columns were washed with Buffer A (20 mM Tris-HCl, pH 8.0, 500 mM NaCl, 20 mM imidazole) to remove nonspecific proteins. His-tagged AARS1 protein was eluted with Buffer B (20 mM Tris-HCl, pH 8.0, 500 mM NaCl, 500 mM imidazole). Eluted fractions were concentrated using Amicon Ultra centrifugal filter units (Sigma-Aldrich), and purified proteins were resuspended in MST buffer (PBS containing 0.05% Tween-20) for downstream assays.

### MST.

Binding affinity between purified proteins and HCY was assessed using the Monolith NT.115 pico (NanoTemper Technologies) according to the manufacturer’s instructions. Briefly, 5 μL of target protein was mixed with 5 μL of serially 2-fold diluted HCY or NaLac and incubated at room temperature for 20 minutes. MST measurements were performed in MST buffer (PBS containing 0.05% Tween-20). Binding curves were analyzed using MO.Affinity Analysis software (NanoTemper) and GraphPad Prism 8.0.1.

### Molecular docking.

The crystal structure of human AARS1 protein (amino acids 1–455; PDB ID: 4XEM) was obtained from the Protein Data Bank (PDB), and the 3D structures of HCY and lactate were retrieved from the PubChem database. AARS1 was preprocessed using the Protein Preparation Wizard in Schrödinger, including removal of water molecules and ligands, addition of hydrogen atoms, peptide bond correction, and energy minimization to ensure structural stability. HCY and lactate was processed using LigPrep to remove ions, generate all possible protonation states and conformers, and perform energy minimization for structural optimization. The enzyme active site and surrounding residues were defined using the Receptor Grid Generation module to create the docking grid box. Molecular docking simulations were performed with the Ligand Docking module to predict the binding modes of HCY within the active site of AARS1. Docking results were visualized and further analyzed using PyMOL software.

### Statistics.

Statistical analyses were performed using GraphPad Prism 8.0.1 software. Quantitative data are presented as mean ± SEM from at least 3 independent experiments. Unpaired 2-tailed Student’s *t* tests were used for comparisons between 2 groups; 2-tailed nested *t* test, 1-way ANOVA, and 2-way ANOVA were performed for comparisons among multiple groups. Statistical significance was set at *P* < 0.05.

### Study approval.

The iPSC lines used in this study included 4 donor-derived lines (ihtc-03, HD40, HD42, and HD66), which were generated and differentiated with both verbal and written informed consent obtained from the respective donors. The IMR90-4 human iPSC line was obtained under a WiCell agreement (no. 17-W0063), and the RC01001-A human iPSC line was provided by Zhongsheng Traceable Biotechnology Co. Ltd. with appropriate authorization. Human stem cell–related procedures were reviewed and approved by the Ethics Committee of Nanjing Medical University. All animal studies were conducted in accordance with institutional guidelines and were approved by the Institutional Animal Care and Use Committee of Nanjing Medical University (protocol no. IACUC2404086).

### Data availability.

The raw data of bulk RNA-Seq and CUT&Tag in this study have been deposited in the NCBI’s Sequence Read Archive (SRA) (accession SRR36984554–SRR36984568, SRR36355749–SRR36355769, and SRR36321738–SRR36321743). Values for all data points in graphs are reported in the Supporting Data Values file.

## Author contributions

XG, YL, and XS conceived, designed, and supervised all the studies and wrote the manuscript. ML, S Wu, and XL conducted the majority of cell culture experiments, differentiated hSOs in vitro, and performed immunostaining and biochemical analyses of hSOs. KL injected AAVs into mice, conducted animal behavioral analyses, and performed biochemical analyses in mice. CL was responsible for mouse maintenance. S Wang and HY analyzed RNA-seq and metabolomics data and performed molecular docking experiments. ZZ and YJ contributed to data analysis.

## Conflict of interest

The authors have declared that no conflicts of interest exist.

## Funding support

- National Natural Science Foundation of China (U23A20429, 82371260, 82325015, and 82530038).
- National Key Research and Development Program of China (2021YFA1101802, 2025YFC3408902, and 2021YFA1101800).

## Supplementary Material

Supplemental data

Unedited blot and gel images

Supplemental table 1

Supplemental table 2

Supplemental table 3

Supplemental table 4

Supplemental table 5

Supplemental table 6

Supplemental table 7

Supplemental table 8

Supplemental table 9

Supplemental table 10

Supplemental table 11

Supporting data values

## Version 1 03/10/2026

In-Press Preview

## Version 2 05/01/2026

Electronic publication
