# Expression of Concern for SHMT2 deficiency disrupts transcriptional regulation through homocysteine-mediated suppression of histone lactylation in Huntington’s disease models

**DOI:** 10.1172/JCI214632

**Published:** 2026-11-16

**Authors:** Mingqin Lu, Kexin Li, Shanshan Wu, Zhilong Zheng, Xinyue Li, Shengda Wang, Hanwen Yu, Chunyue Liu, Yueqing Jiang, Xueqin Song, Yan Liu, Xing Guo

Original citation: *J Clin Invest*. 2026;136(9):e196094. https://doi.org/10.1172/JCI196094

Citation for this expression of concern: *J Clin Invest*. 2026;136(22):e214632. https://doi.org/10.1172/JCI214632

The authors recently alerted the Editors to errors in the source data and figures for Figure 2G and Supplemental Figures 3A, 5A, and 5G that were identified in response to an institutional mandate for a post-publication audit of the data. Although the authors have indicated that independent lines of investigation support the article’s conclusions, the Editors are issuing this Expression of Concern to alert readers to the issues. We have requested institutional oversight into this matter and will alert readers to the outcome when the institutional evaluation is complete.

## Version 1 11/16/2026

Electronic publication

