# Supplementary material for: SHMT2 deficiency disrupts transcriptional regulation through homocysteine-mediated suppression of histone lactylation in Huntington’s disease models
Source: J Clin Invest. 2026 Mar 10;136(9):e196094. doi: 10.1172/JCI196094 (PMC13132398; doi:10.1172/JCI196094)
Supplement: Supplemental data [file jci-136-196094-s111.pdf]

SHMT2 deficiency disrupts transcriptional regulation through homocysteine-mediated suppression of histone lactylation in Huntington's Disease models

Mingqin Lu[1,2], Kexin Li[1], Shanshan Wu[3], Zhilong Zheng[1], Xinyue Li[1], Shengda Wang[1], Hanwen Yu[3], Chunyue Liu[1], Yueqing Jiang[1], Xueqin Song[4,5], Yan Liu[2,3], Xing Guo[1,2,6]

1. Department of Neurobiology, School of Basic Medical Sciences, Nanjing Medical University, Nanjing, Jiangsu, China.
2. State Key Laboratory of Reproductive Medicine and Offspring Health, Nanjing Medical University, Nanjing, Jiangsu, China.
3. Institute for Stem Cell and Neural Regeneration, School of Pharmacy, Nanjing Medical University, Nanjing, Jiangsu, China.
4. Key Laboratory of Clinical Neurology, Ministry of Education, Hebei Medical University, Shijiazhuang, Hebei, China.
5. Department of Neurology, Key Neurological Laboratory of Hebei Province, the Second Hospital of Hebei Medical University, Shijiazhuang, Hebei, China.
6. Jiangsu Key Laboratory of Molecular Targets and Intervention for Metabolic Diseases, Nanjing Medical University, Nanjing, Jiangsu, China.

Authorship note: MQL, KXL, SSW and ZLZ contributed equally to this work.

Declaration of interests

The authors have declared that no conflicts of interest exist.

Address correspondence to: Xing Guo, Department of Neurobiology, School of Basic Medical Sciences, State Key Laboratory of Reproductive Medicine and Offspring Health, Nanjing Medical University, 101 Longmian Avenue, Nanjing, Jiangsu 211166, China. Phone: +86 25 86869345. E-mail: guox@njmu.edu.cn (XG). Or to: Yan Liu, Institute for Stem Cell and Neural Regeneration, State Key Laboratory of Reproductive Medicine and Offspring Health, School of Pharmacy, Nanjing Medical University, 101 Longmian Avenue, Nanjing, Jiangsu 211166, China. Phone: +86 25 86868478. E-mail: yanliu@njmu.edu.cn (YL). Or to: Xueqin Song, Department of Neurology, Key Laboratory of Clinical Neurology, Ministry of Education, the Second Hospital of Hebei Medical University, 215 Heping West Road, Shijiazhuang, Hebei 050000, China. Phone: +86 0311-66003955. Email: 27100829@hebmu.edu.cn (XQS).

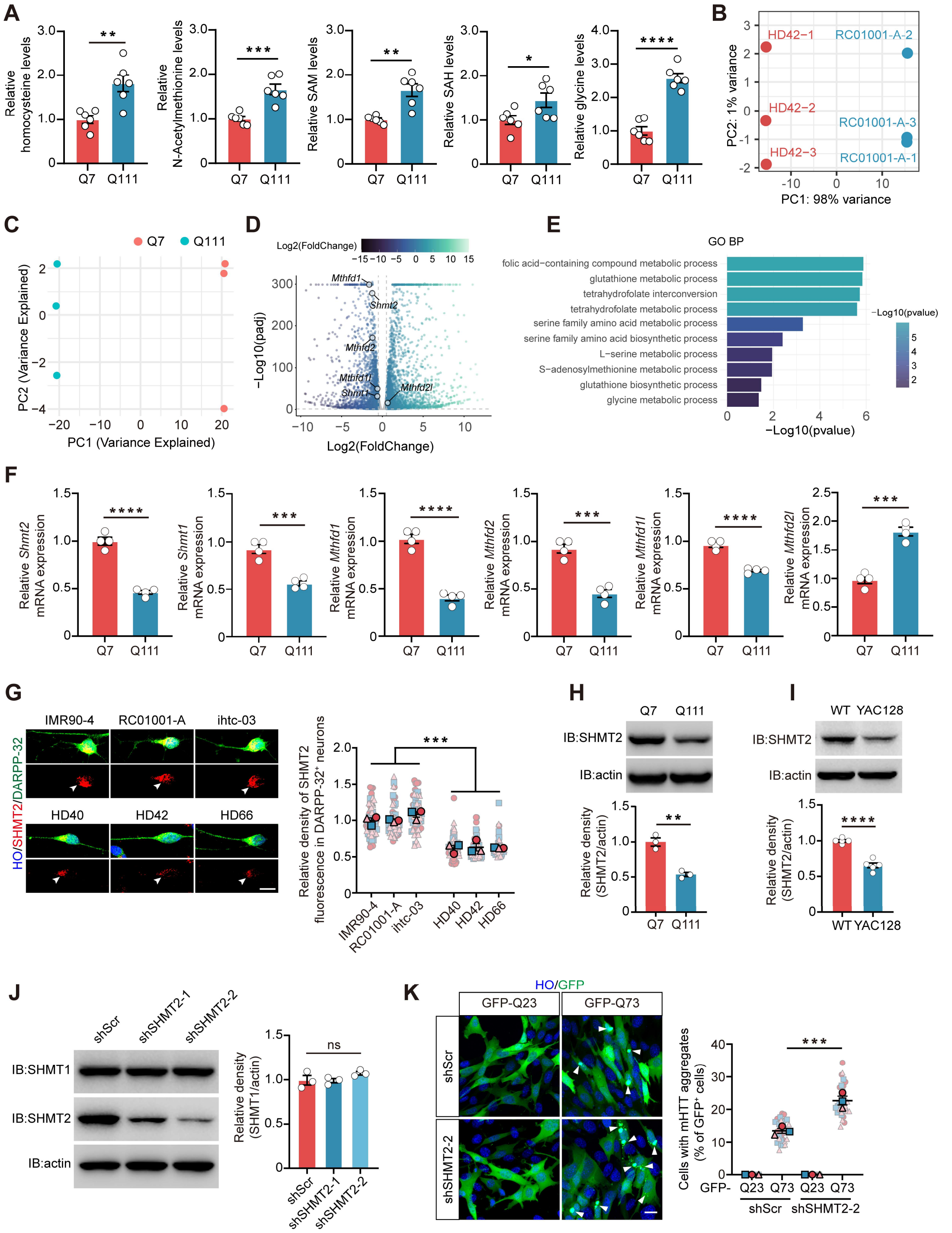

Supplemental Figure 1. Multi-omics analysis reveals 1C metabolism dysregulation and reduced SHMT2 expression in HD models. (A) Relative levels of metabolites related to one-carbon metabolism, including HCY, N-acetylmethionine, SAM, SAH, and glycine, in HdhQ7 and HdhQ111 cells (n = 6). **(B)** PCA of bulk RNA-seq data from Con-hSOs (derived from RC01001-A) and HD-hSOs (derived from HD42). **(C)** PCA of bulk RNA-seq data from HdhQ7 and HdhQ111 cells. **(D)** Volcano plot of DEGs between HdhQ111 and HdhQ7 cells. Key one-carbon metabolism–related genes are

labeled. DEG cutoff: |log2FC| ≥ 0.25, padj ≤ 0.05. **(E)** GO Biological Process enrichment analysis of DEGs from panel D, ranked by significance ($-Log_{10}$(pvalue)). **(F)** Relative mRNA levels of genes related to one-carbon metabolism in HdhQ7 and HdhQ111 cells (n = 4). **(G)** SHMT2 immunofluorescence in control and HD iPSC-derived DARPP-32$^+$ neurons (n = 3; scale bar, 10 μm). **(H)** Representative immunoblot showing SHMT2 protein levels in HdhQ7 and HdhQ111 cells (n = 3). **(I)** Immunoblot analysis of SHMT2 protein levels in striatal tissues from 4-month-old WT and HD transgenic YAC128 mice (n = 5). **(J)** Immunoblot analysis and quantification of SHMT1 and SHMT2 expression in HdhQ7 cells transduced with shScr, shSHMT2-1, or shSHMT2-2 (n = 3). **(K)** PolyQ aggregates in control and SHMT2-knockdown HdhQ7 cells expressing GFP-HTTEx1-Q23 or GFP-HTTEx1-Q73; aggregates in GFP$^+$ cells were quantified (scale bar, 20 μm; n = 3).

Data are mean ± SEM. Unpaired Student's t-test was used for panels A, F, H, and I; nested t-test for panel G; and one-way ANOVA with Tukey's test for panel J and K. *P < 0.05, **P < 0.01, ***P < 0.001, ****P < 0.0001.

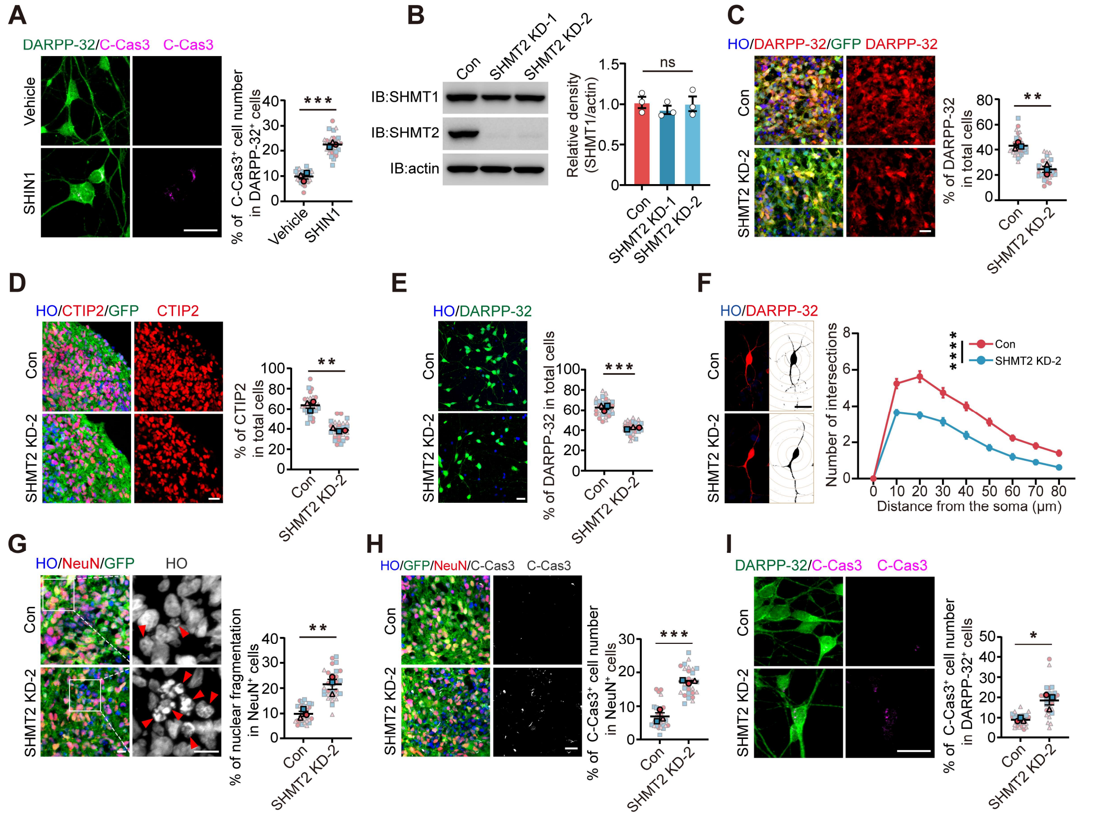

Supplemental Figure 2. Loss of SHMT2 induces neuronal degeneration in iPSCs-derived hSOs. (A) Cleaved caspase-3 immunofluorescence in neurons dissociated from D 50 Con-hSOs and treated with 10 μM SHIN1 for 48 h (n = 3; scale bar, 20 μm). **(B)** Immunoblot of SHMT2 and SHMT1 in iPSCs transduced with SHMT2-targeting CRISPRi sgRNA or control sgRNA (n = 3). **(C)** DARPP-32 and GFP co-staining in control and SHMT2 KD hSOs at D 60; DARPP-32$^+$ cells quantified as shown in scatter plots (scale bar, 20 μm; n = 3). **(D)** CTIP2 and GFP immunostaining in control and SHMT2 KD hSOs at D 60. The proportion of CTIP2$^+$ cells is shown in the accompanying scatter plots (scale bar, 20 μm; n = 3). **(E)** DARPP-32 immunostaining in neurons dissociated from control and SHMT2 KD hSOs at D 60; DARPP-32$^+$ cell fractions were quantified (scale bar, 20 μm; n = 3). **(F)** Morphological analysis of neurons dissociated from control and SHMT2 KD hSOs at D 60 using Sholl quantification (scale bar, 20 μm; n = 60 neurons per group). **(G)** Nuclear fragmentation in control and SHMT2 KD hSOs at D 60 (scale bar, 10 μm; n = 3). **(H)** Cleaved caspase-3 immunofluorescence in control and SHMT2 KD hSOs at D 60 (scale bar, 20 μm; n = 3). **(I)** Cleaved caspase-3 immunofluorescence in neurons dissociated from control and SHMT2 KD hSOs at D 60 (scale bar, 20 μm; n = 3).

Data are presented as mean ± SEM. Unpaired Student's t-test was used in A, C–E, and G–I; one-way ANOVA with Tukey's test for panel B; two-way ANOVA with Sidak's test was used in F. $^*P < 0.05$, $^{**}P < 0.01$, $^{***}P < 0.001$, $^{****}P < 0.0001$.

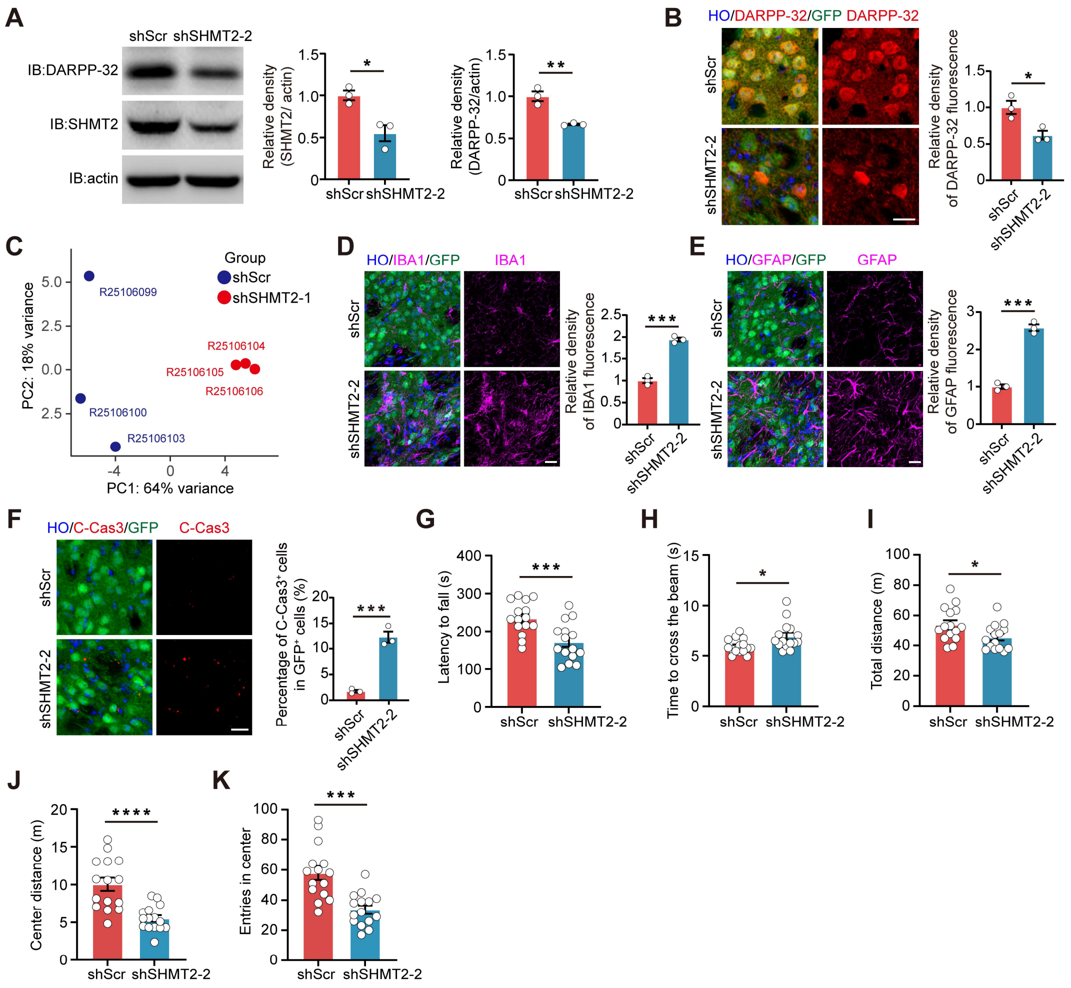

Supplemental Figure 3. SHMT2 deficiency induces neurodegeneration and motor dysfunction in vivo. (A) SHMT2 knockdown validation showing reduced SHMT2 and DARPP-32 levels in mouse striatum (n = 3). **(B)** DARPP-32 and GFP immunofluorescence in the striatum of WT mice injected with shScr or shSHMT2 (n = 3; scale bar, 20 μm). **(C)** PCA of RNA-seq data from striata of WT mice injected with shScr or shSHMT2. **(D)** Immunofluorescence of IBA1 (magenta) and GFP (green) in the striatum of WT mice injected with AAV-shScr or AAV-shSHMT2 (n = 3 per group; scale bar, 20 μm). **(E)** Immunofluorescence of GFAP (magenta) and GFP (green) in the striatum of WT mice injected with AAV-shScr or AAV-shSHMT2 (n = 3 per group; scale bar, 20 μm). **(F)** Cleaved caspase-3 and GFP staining in the striatum of WT mice injected with shScr or shSHMT2, with quantification of cleaved caspase-3^{+} cells among GFP^{+} cells (n = 3 per group; scale bar, 20 μm). **(G–K)** Behavioral analyses of WT mice injected with AAV-shScr or AAV-shSHMT2 at 2 months of age and tested at 4 months (n = 15 per group), including rotarod (G), beam-crossing (H), and open-field assays (I–K).

Data are shown as mean ± SEM. Unpaired Student's t-test was used in A–B and D–K. *P < 0.05, **P < 0.01, ***P < 0.001, ****P < 0.0001.

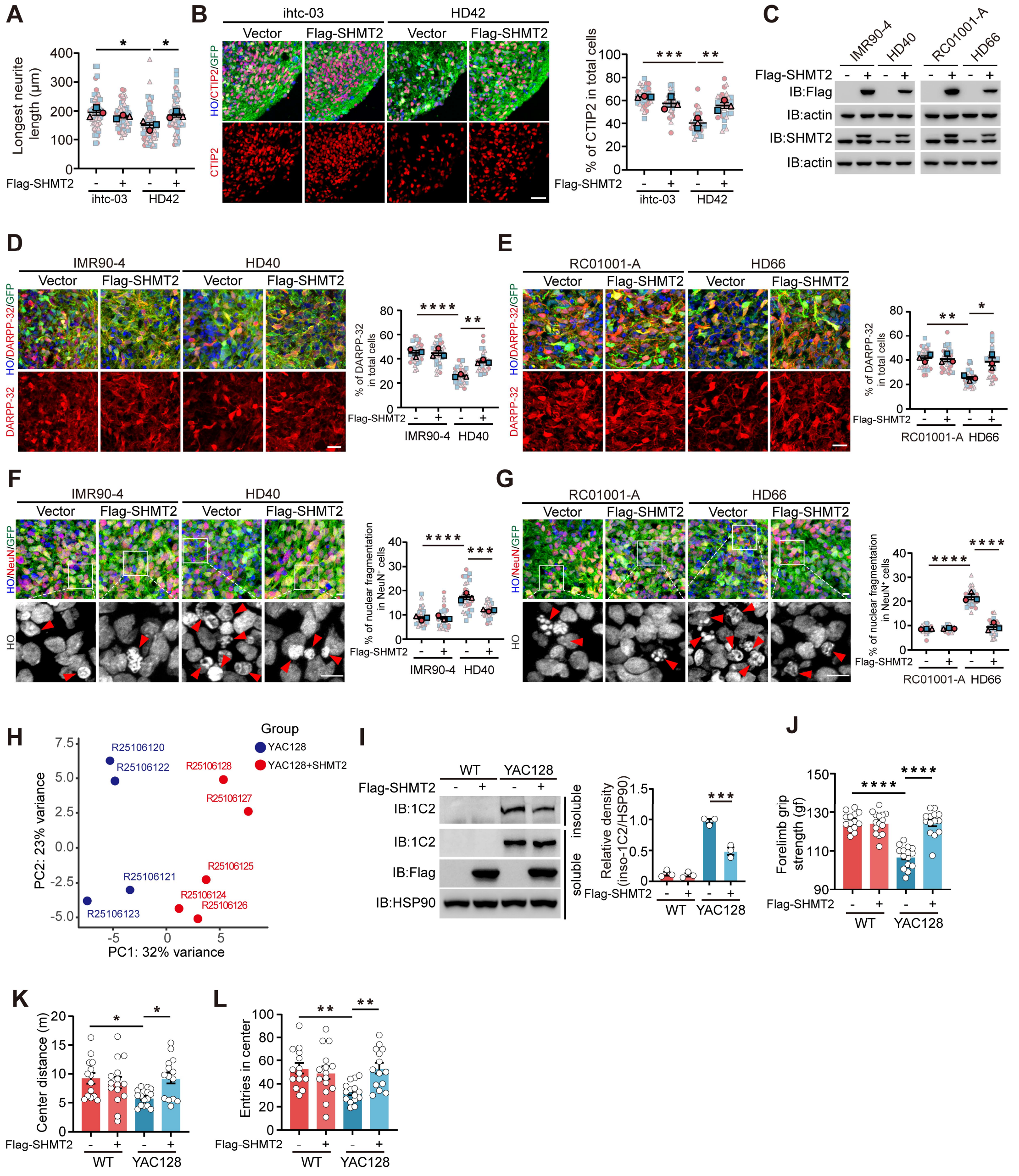

Supplemental Figure 4. SHMT2 overexpression ameliorates neurodegeneration both in vivo and in vitro. (A) Quantification of longest neurite length in neurons derived from control and SHMT2-overexpressing con-hSOs and HD-hSOs (n = 3). **(B)** Immunofluorescence of CTIP2 (red) and GFP (green) in control and SHMT2-overexpressing con-hSOs and HD-hSOs at D 60, with quantification of the proportion of CTIP2$^+$ cells (n = 3; scale bar, 20 µm). **(C)** Western blot validation of SHMT2 overexpression in control and HD iPSCs transduced with control vector or FLAG-SHMT2. **(D–E)** Representative immunofluorescence images showing DARPP-32 and GFP co-staining in control-hSOs (IMR90-4 and RC01001-A) and HD-hSOs (HD40 and HD66) expressing control vector or FLAG-SHMT2 at D 60, with quantification of DARPP-32$^+$ cells (scale bar, 20 µm; n = 3). **(F–G)** Immunofluorescence analysis showing nuclear fragmentation in control (IMR90-4 and RC01001-A) and HD (HD40

and HD66) hSOs expressing GFP or GFP–SHMT2 at D 60, with quantification of cells exhibiting fragmented nuclei (scale bar, 10 μm; n = 3). **(H)** PCA of RNA-seq transcriptomic profiles from the striata of YAC128 mice injected with AAV-control or AAV-SHMT2. **(I)** Detergent-insoluble mHTT species in striatal lysates from WT and YAC128 mice with or without AAV-SHMT2 overexpression were assessed by immunoblotting using the polyQ-specific antibody 1C2 (n = 3). **(J–L)** Behavioral tests performed 2 months after AAV-Con or AAV-SHMT2 injection in 4-month-old mice, assessing grip strength (J), center distance traveled (K), and center entries (L) (n = 14–15).

Data are shown as mean ± SEM. One-way ANOVA followed by Tukey's multiple comparisons test was used in A, B, D-G and I-L. *P < 0.05, **P < 0.01, ***P < 0.001, ****P < 0.0001.

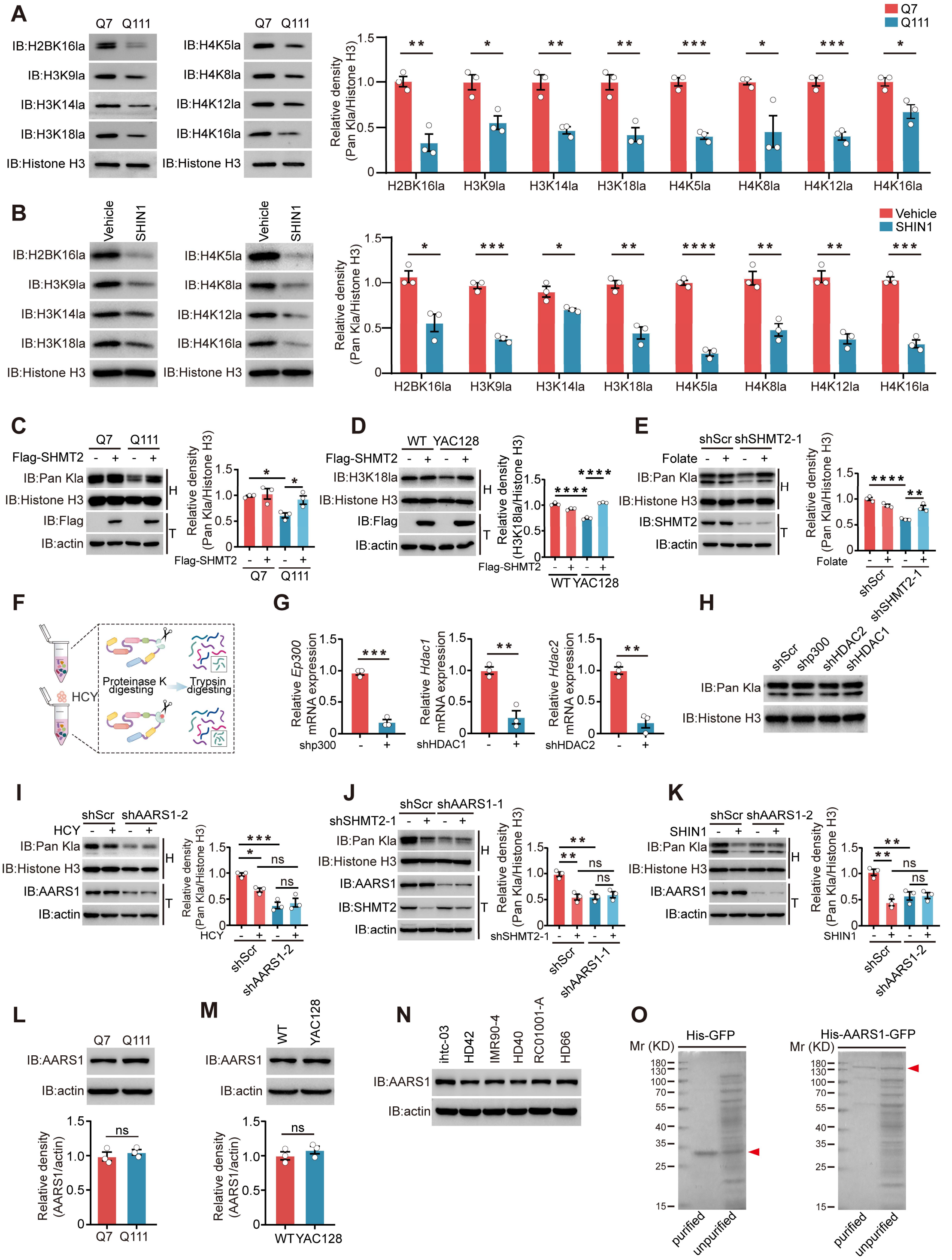

Supplemental Figure 5. HCY suppresses histone lactylation through AARS1. (A and B) Site-specific histone lactylation was detected by immunoblotting in HdhQ7/HdhQ111 cells (A) and in SHIN1-treated HdhQ7 cells (B) (n = 3). **(C)** Pan-histone lactylation in HdhQ7 and HdhQ111 cells expressing control vector or Flag-SHMT2 (n = 3). **(D)** H3K18la levels were determined by immunoblotting in empty vector or Flag-SHMT2-transduced WT and YAC128 mice (n = 3). **(E)** Pan-histone lactylation levels were analyzed in control and SHMT2-knockdown HdhQ7 cells,

treated with or without folate (100 μM, 48 h) (n = 3). **(F)** Workflow of Limited Proteolysis Mass Spectrometry (LiP-MS) analysis. **(G)** Relative mRNA levels of *Ep300*, *Hdac1*, and *Hdac2* in control and knockdown HdhQ7 cells (n = 3). **(H)** Pan-histone lactylation in control, p300-, HDAC1-, and HDAC2-knockdown HdhQ7 cells. **(I)** Histone lactylation was assessed in control and AARS1-knockdown HdhQ7 cells, treated with or without HCY (500 μM, 48 h) (n = 3). **(J)** Pan-histone lactylation was analyzed in control and AARS1-knockdown HdhQ7 cells, each treated with or without shSHMT2 (n = 3). **(K)** Pan-histone lactylation was analyzed in control and AARS1-knockdown HdhQ7 cells with or without SHIN1 (10 μM, 48 h) (n = 3). **(L)** AARS1 protein levels were determined in HdhQ7 and HdhQ111 cells (n = 3). **(M)** AARS1 protein levels were assessed in striatal tissue from 4-month-old WT and YAC128 mice (n = 3). **(N)** AARS1 protein levels were assessed in hSOs derived from HD (HD40, HD42, HD66) and control (ihtc-03, IMR90-4, RC01001-A) iPSCs. **(O)** Coomassie-stained SDS-PAGE of purified proteins.

Data are presented as mean ± SEM. Unpaired Student's t-test was used for A, B, G, L, and M; one-way ANOVA followed by Tukey's multiple comparisons test was used for C–E, and I–K. *$P < 0.05$, **$P < 0.01$, ***$P < 0.001$, ****$P < 0.0001$.

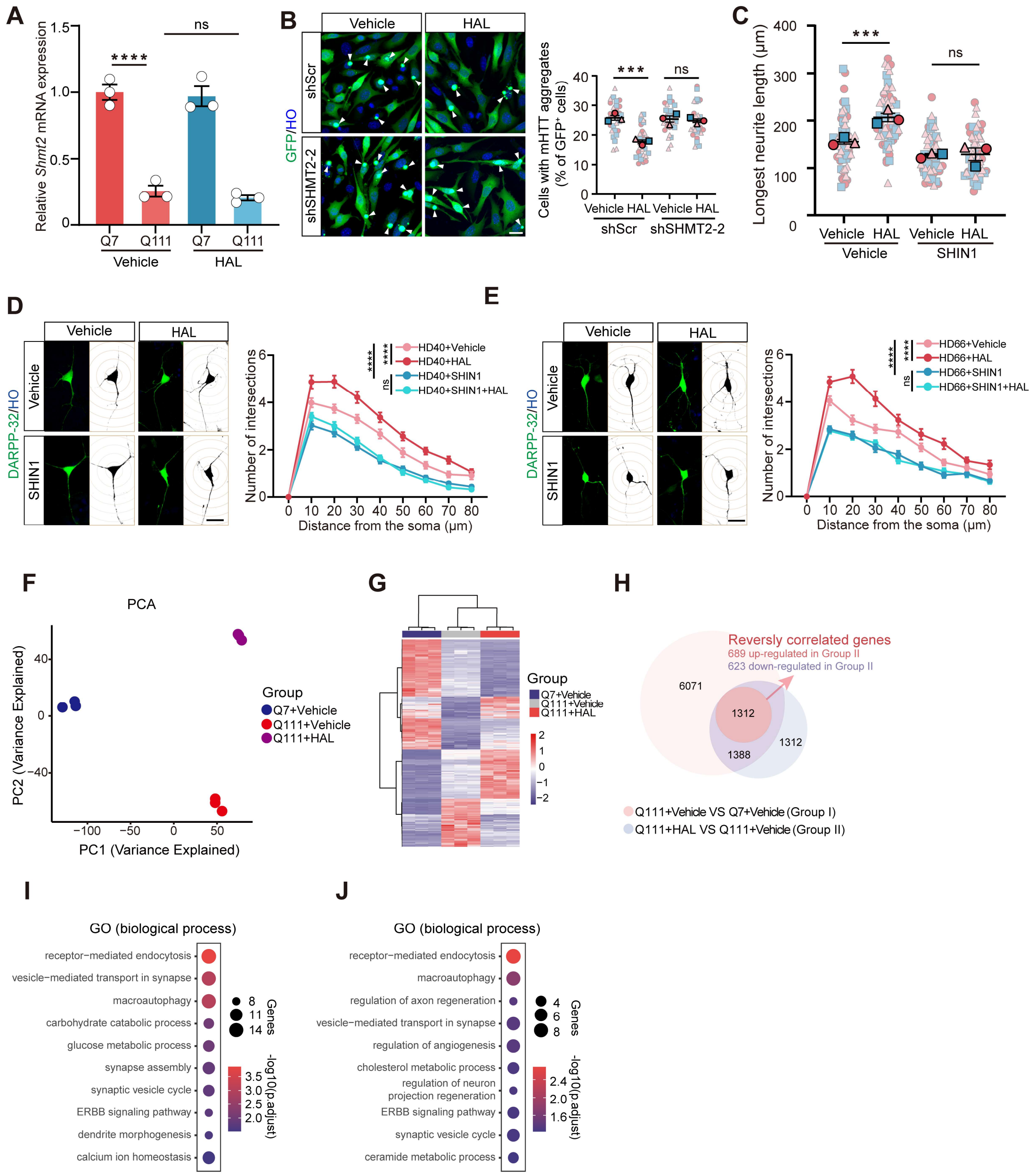

Supplemental Figure 6. Haloperidol alleviates metabolic-epigenetic dysregulation via SHMT2-dependent mechanisms. (A) Relative mRNA levels of *Shmt2* genes in HdhQ7 and HdhQ111 cells treated with haloperidol (20 μM) for 48 hours (n = 3). **(B)** Representative images of HdhQ111 cells expressing GFP-HTTEx1-Q73 treated with vehicle or haloperidol, with or without SHMT2 knockdown. PolyQ aggregates (white arrows) were quantified (scale bar, 20 μm; n = 3). **(C)** Quantification of the longest neurite length in HD-hSOs treated with or without SHIN1 and subsequent haloperidol treatment (n = 3). **(D and E)** Representative images of DARPP-32–positive neurons (green) counterstained with Hoechst (blue) derived from HD-hSOs (HD40 and HD66) treated with or without SHIN1 followed by haloperidol. Neuronal complexity was quantified by Sholl analysis (scale bar, 20 μm; n = 60). **(F)** Principal component analysis (PCA) of bulk RNA-seq data from HdhQ7 + Vehicle, HdhQ111 + Vehicle and HdhQ111+HAL cells. **(G)** Heatmap of markedly altered intersecting genes between

Q111 + Vehicle vs. Q7 + Vehicle and Q111+HAL vs. Q111 + Vehicle. **(H)** Venn diagram of altered genes between Q111 + Vehicle vs. Q7 + Vehicle and Q111+HAL vs. Q111 + Vehicle. Genes with negatively correlated expression changes are highlighted (n = 1312). **(I and J)** GO biological process enrichment analysis of concordant genes identified by integrated CUT&Tag and RNA-seq analysis, as shown in Figure 7, I and J.

Data are presented as mean ± SEM. One-way ANOVA followed by Tukey's multiple comparisons test was used in A, B, and C; two-way ANOVA followed by Sidak's multiple comparisons test was used in D and E. ***P < 0.001 and ****P < 0.0001.
