# Supplementary material for: SHMT2 deficiency disrupts transcriptional regulation through homocysteine-mediated suppression of histone lactylation in Huntington’s disease models
Source: J Clin Invest. 2026 Mar 10;136(9):e196094. doi: 10.1172/JCI196094 (PMC13132398; doi:10.1172/JCI196094)

Full unedited blot for Figure 1

Figure 1G

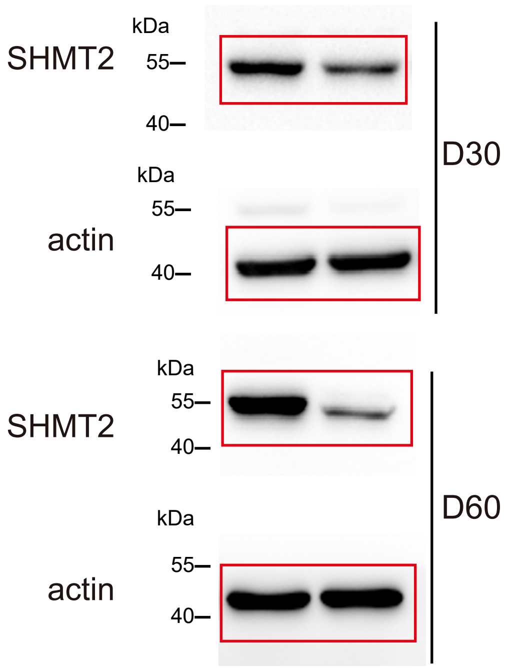

Figure 1H

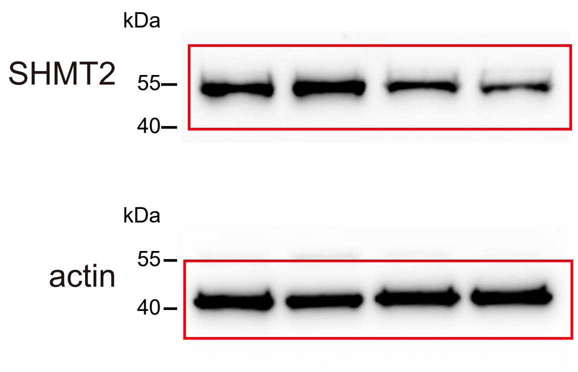

Figure 1I

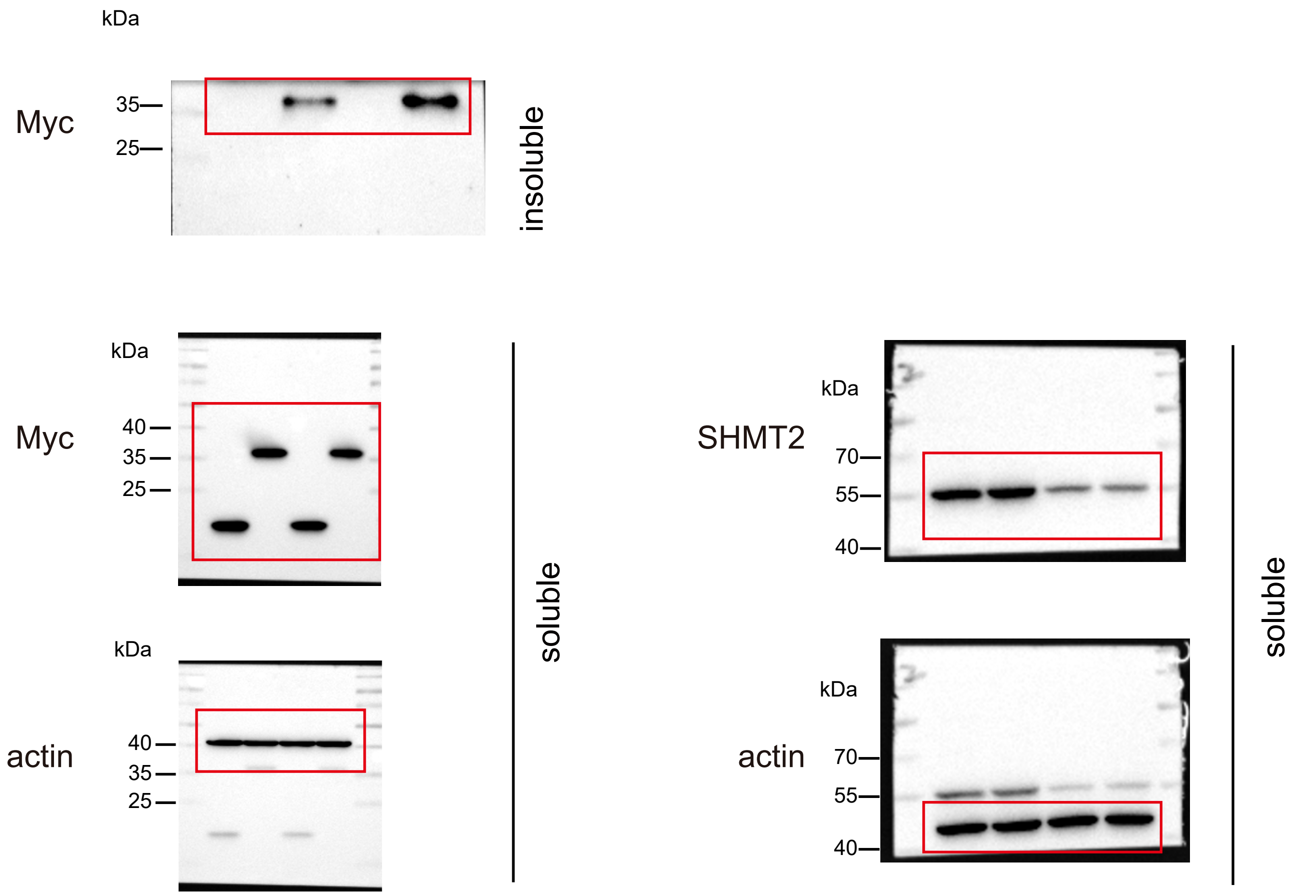

Full unedited blot for Figure 2

Figure 2I

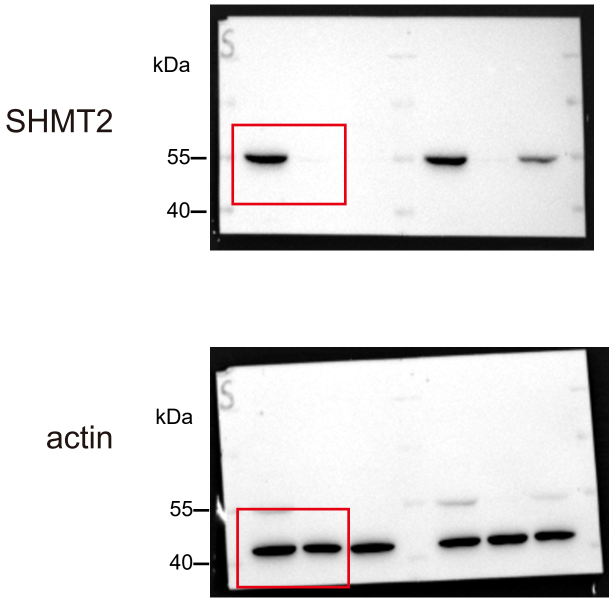

Full unedited blot for Figure 3

Figure 3B

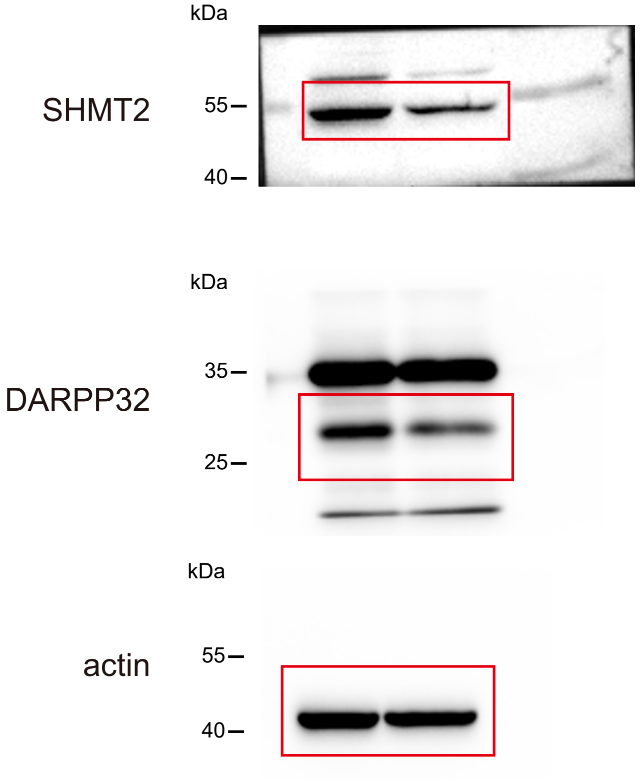

Full unedited blot for Figure 4

Figure 4A

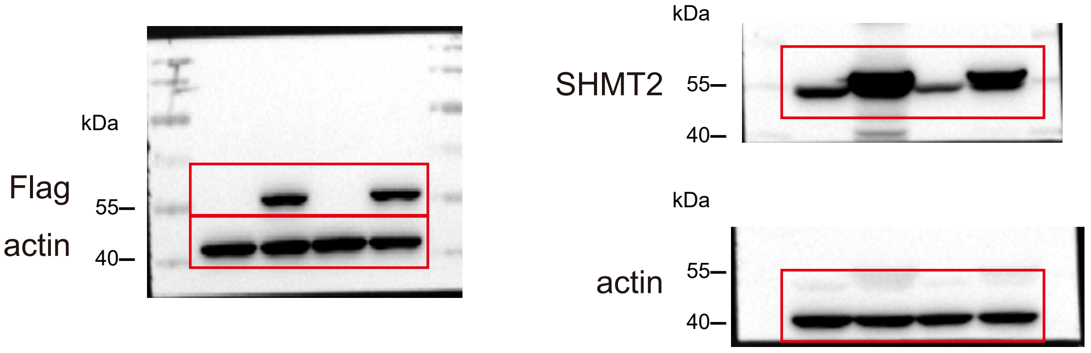

Figure 4H

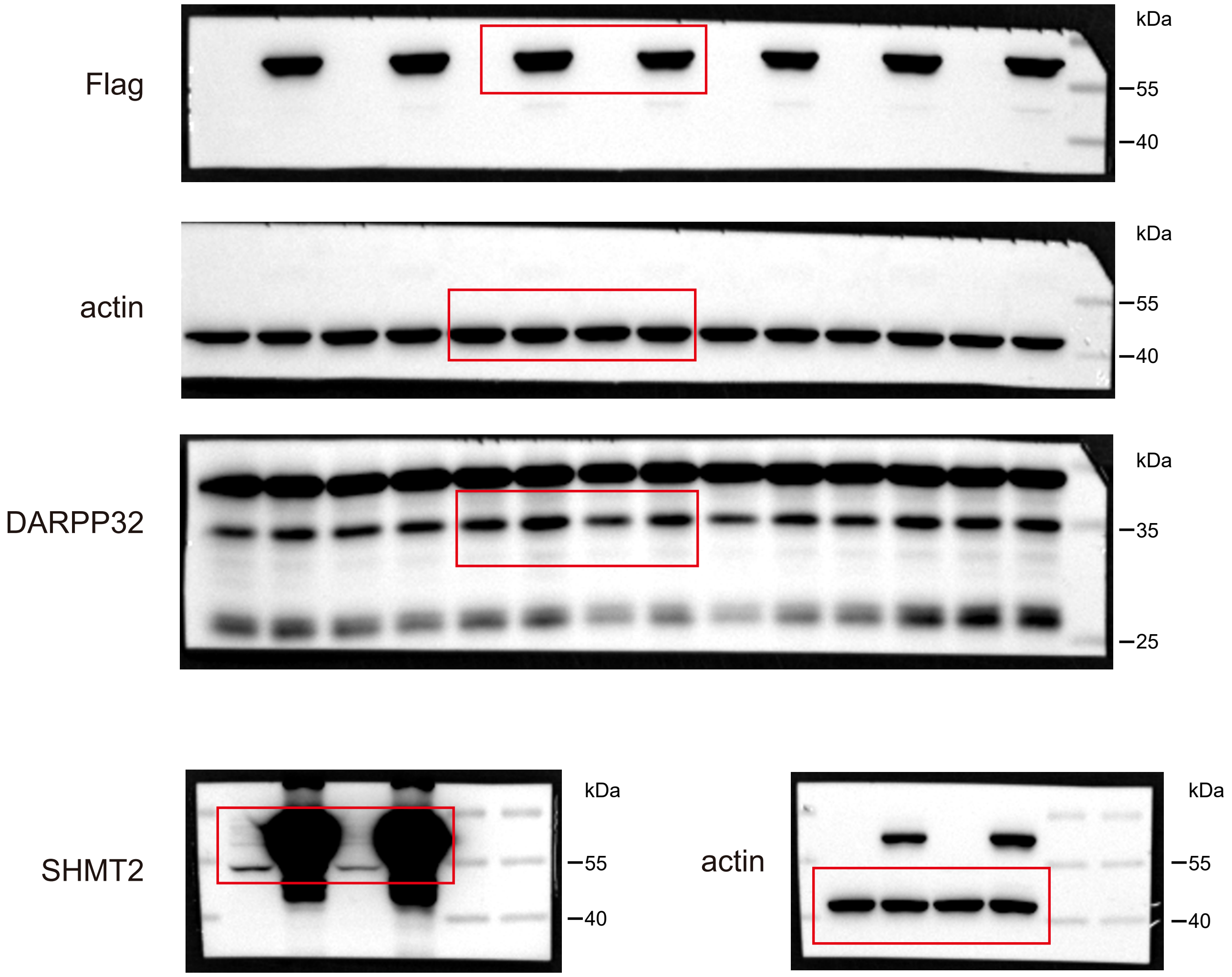

Full unedited blot for Figure 6

Figure 6A

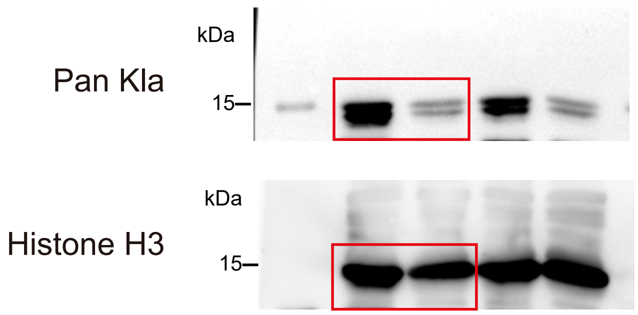

Figure 6B

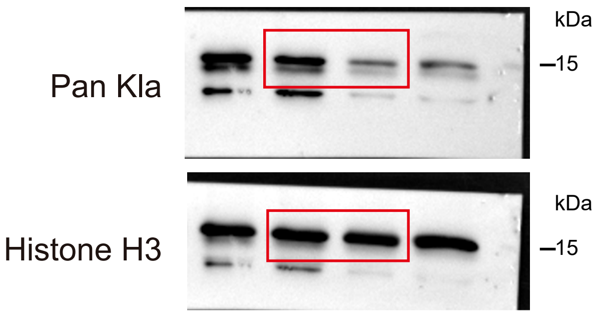

Figure 6C

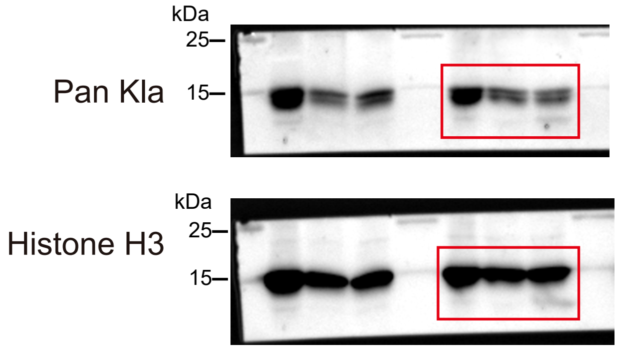

Figure 6D

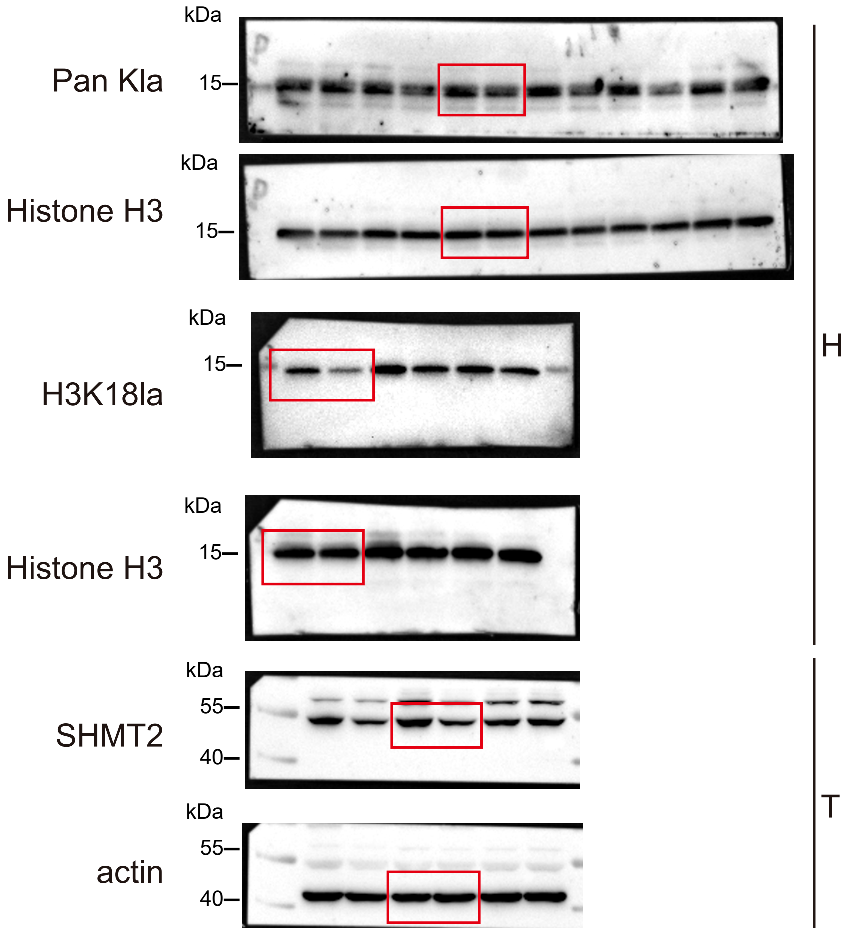

Figure 6E

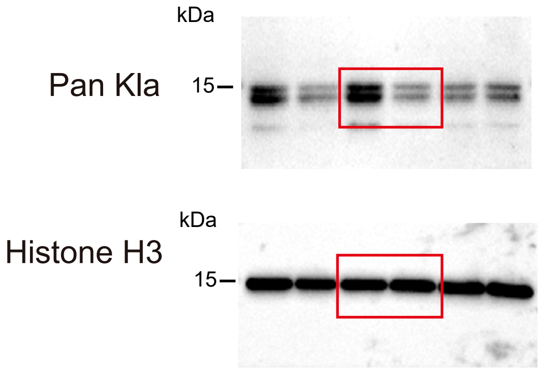

Figure 6F

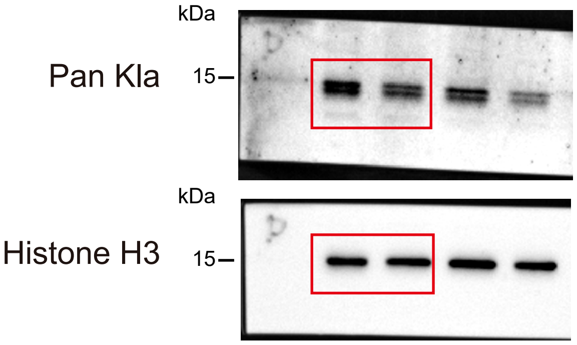

Full unedited blot for Figure 6

Figure 6G

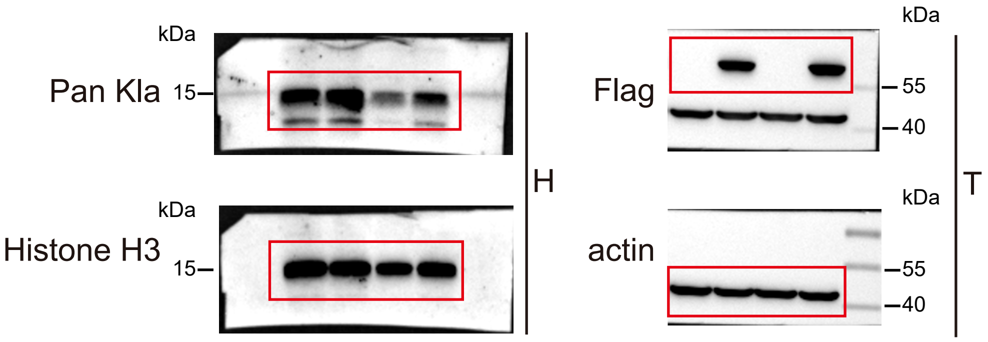

Figure 6H

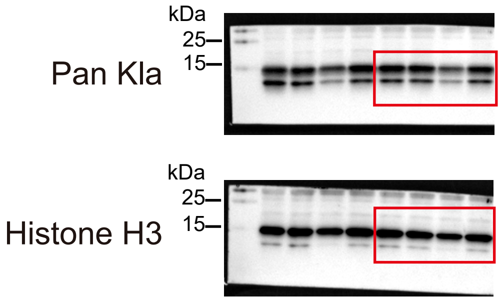

Figure 6J

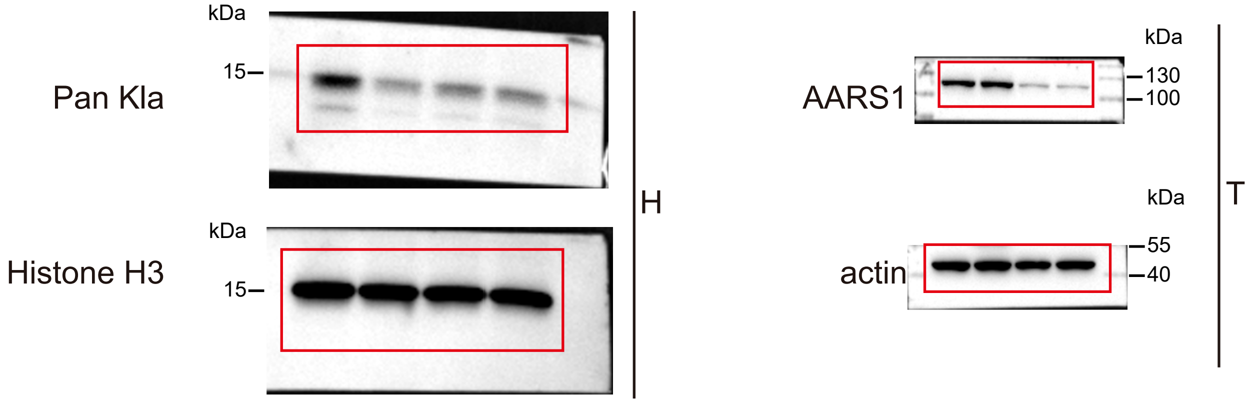

Figure 6K

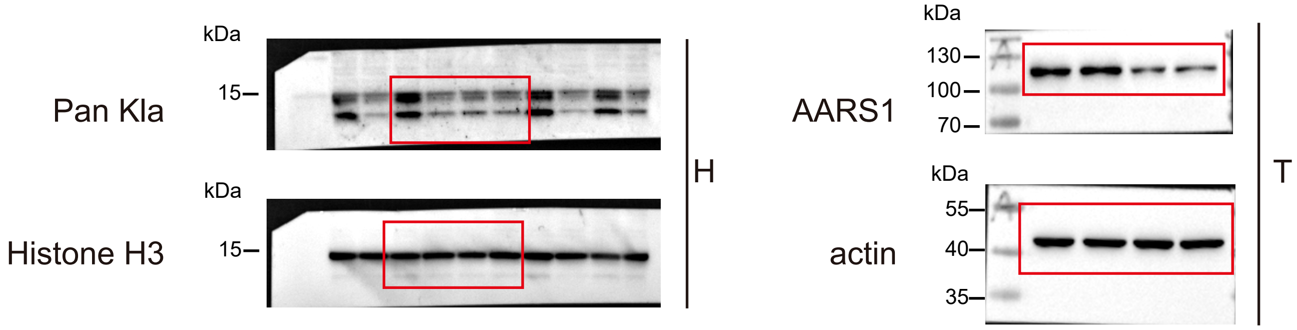

Figure 6L

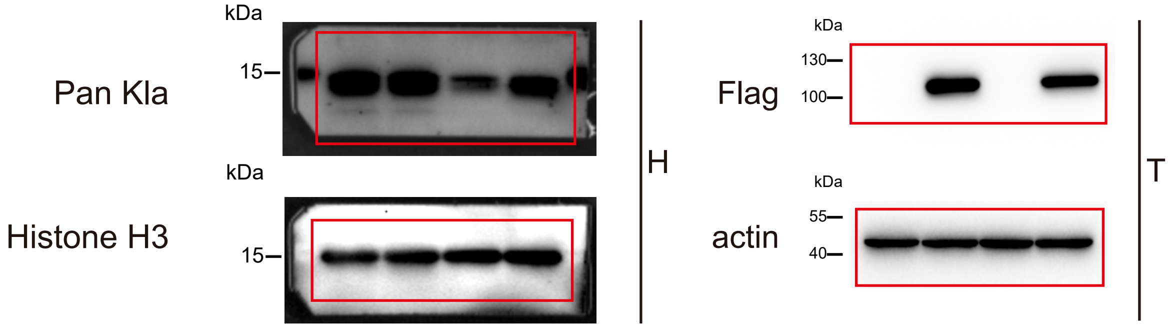

Full unedited blot for Figure 7

Figure 7A

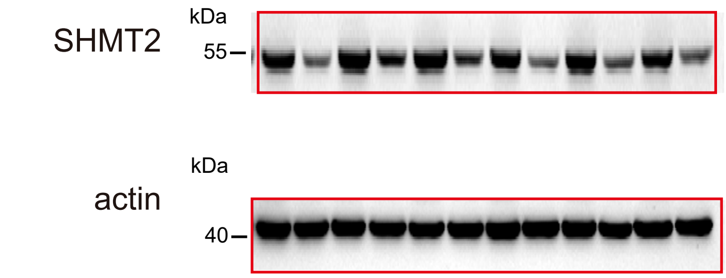

Figure 7C

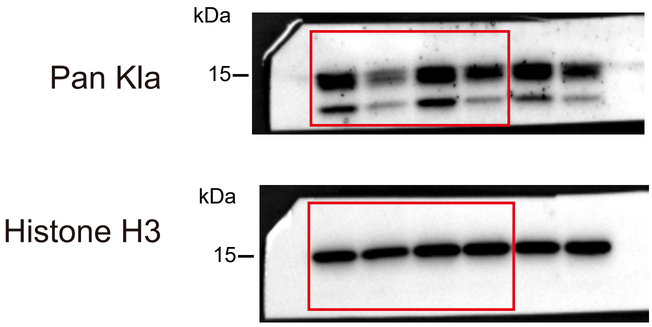

Figure 7D

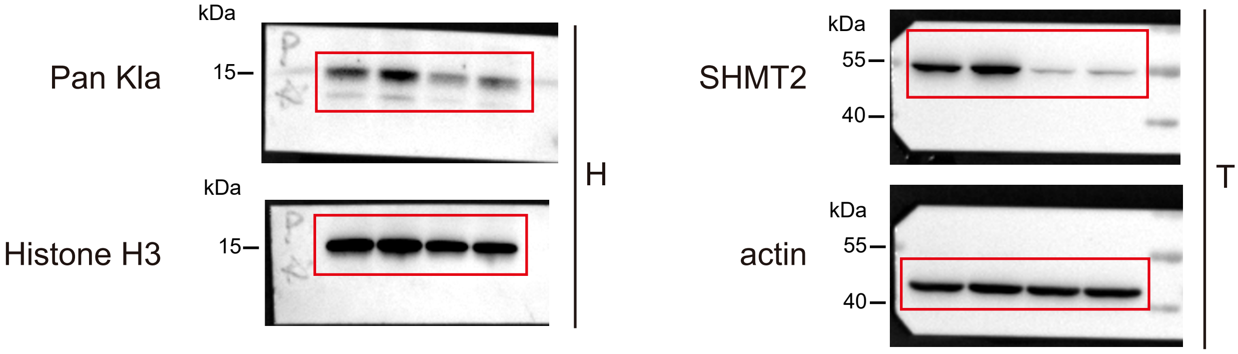

Full unedited blot for Supplemental Figure 1

Figure S1H

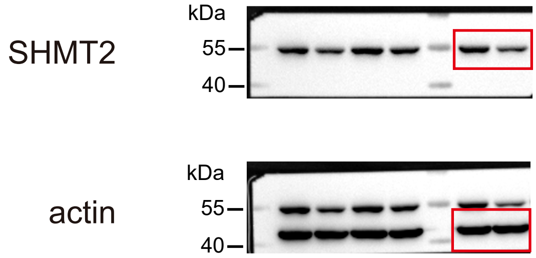

Figure S1I

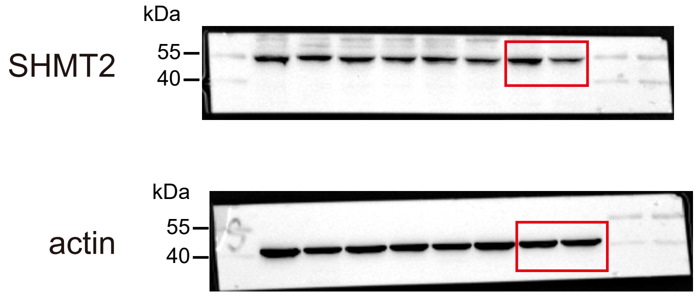

Figure S1J

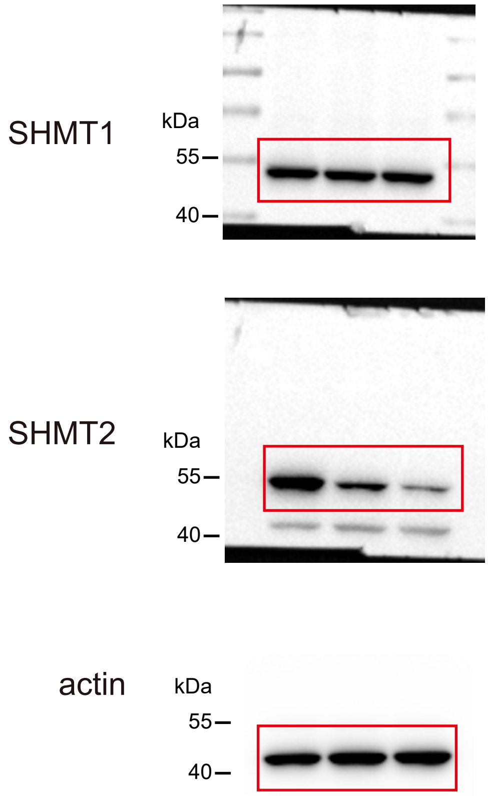

Full unedited blot for Supplemental Figure 2

Figure S2B

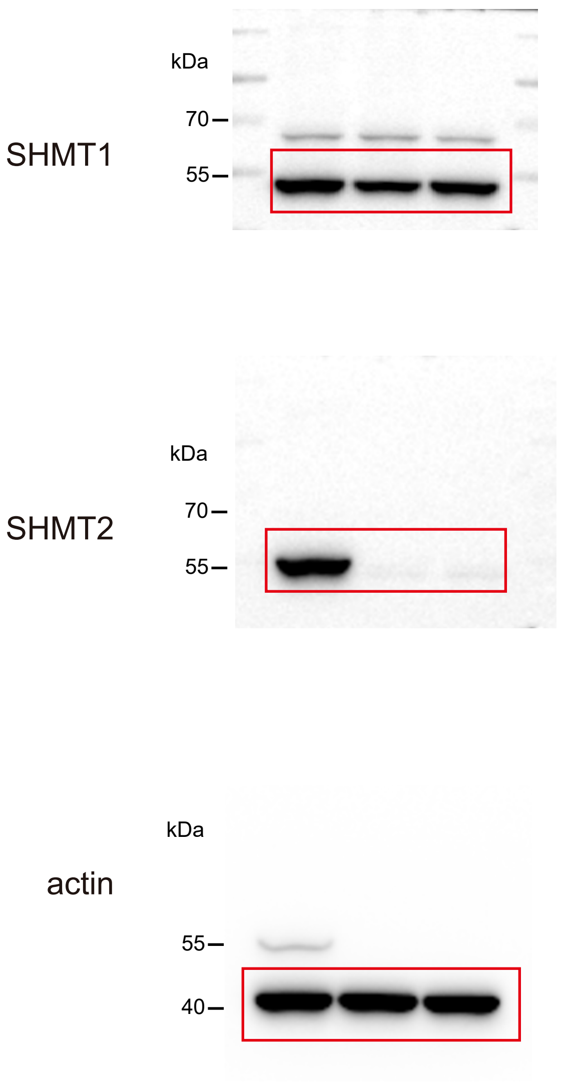

Full unedited blot for Supplemental Figure 3

Figure S3A

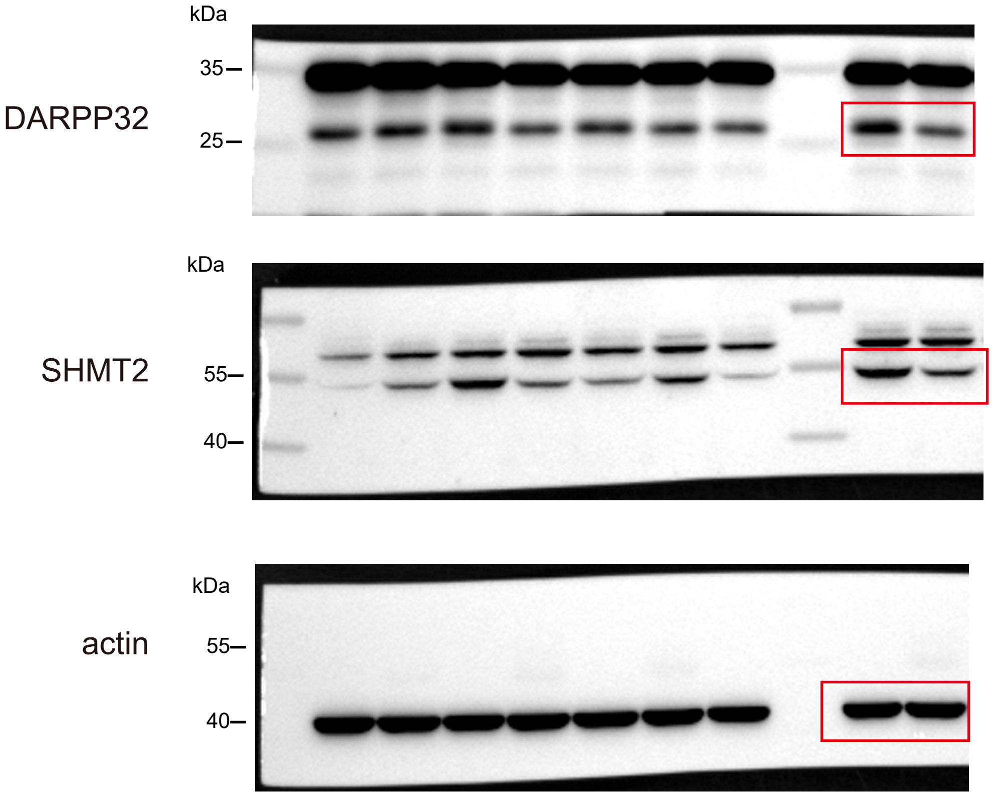

Full unedited blot for Supplemental Figure 4

Figure S4C

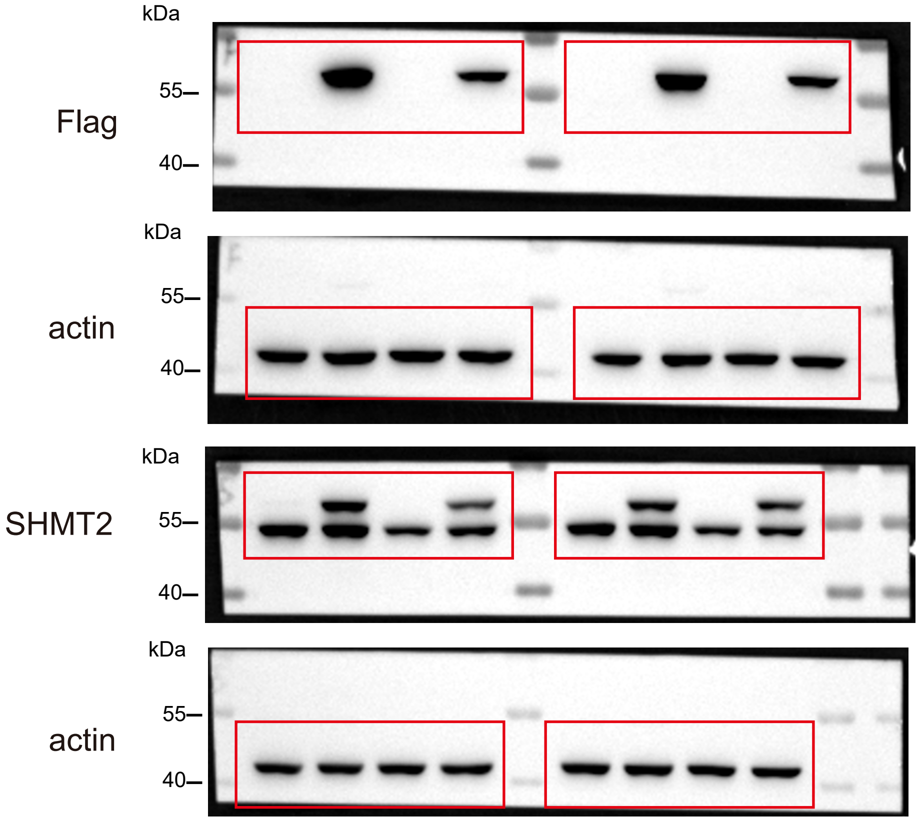

Figure S4I

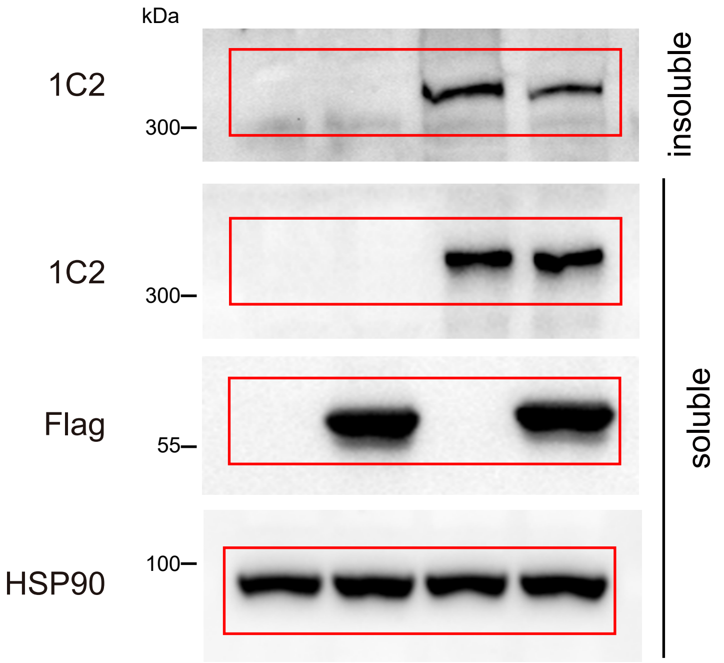

Full unedited blot/gel for Supplemental Figure 5

Figure S5A

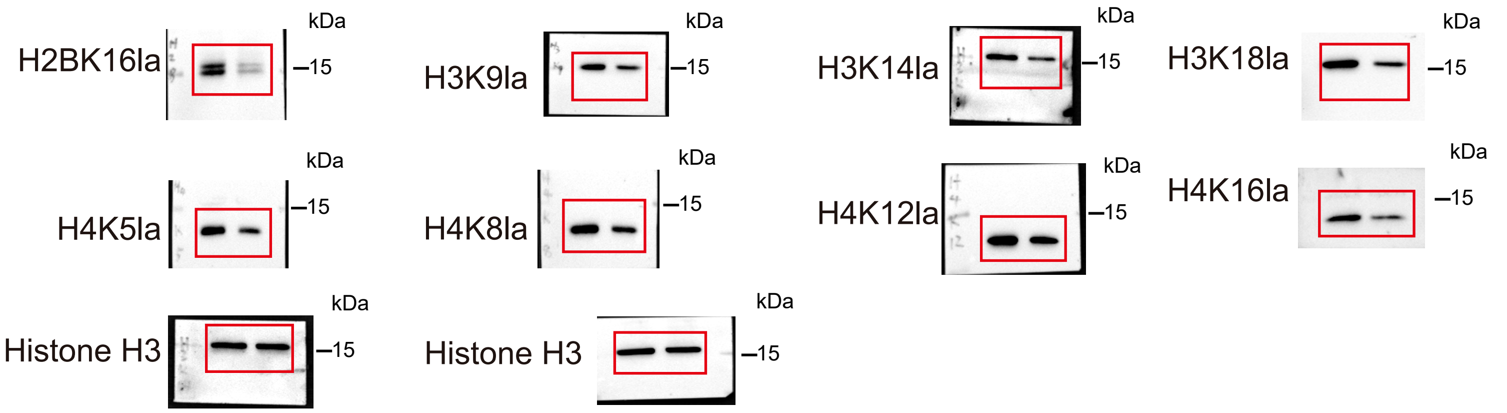

Figure S5B

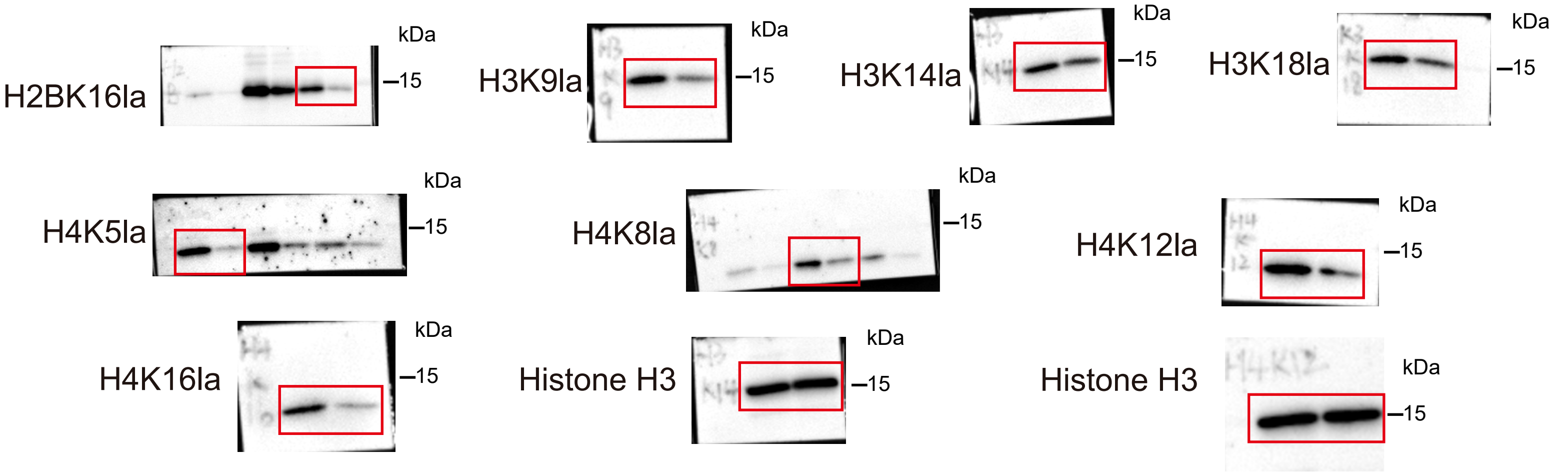

Figure S5C

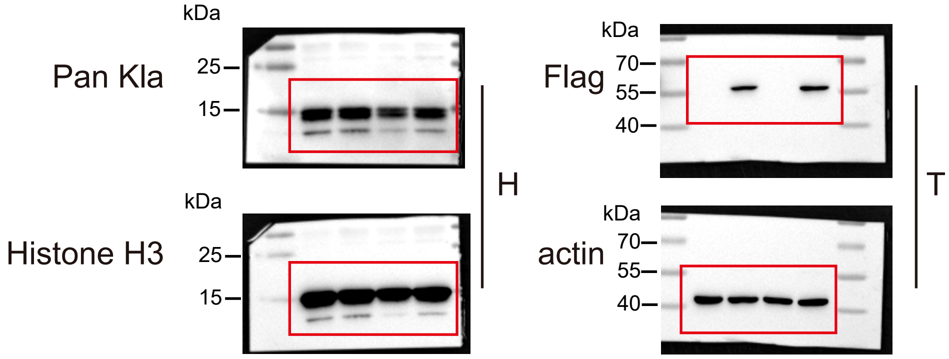

Figure S5D

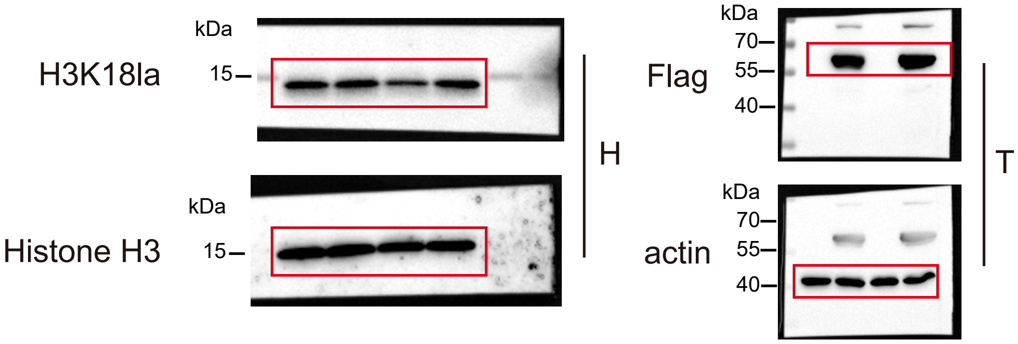

Full unedited blot/gel for Supplemental Figure 5

Figure S5E

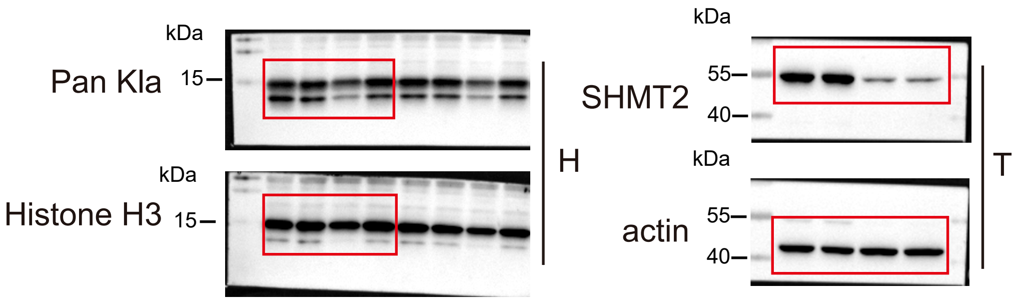

Figure S5H

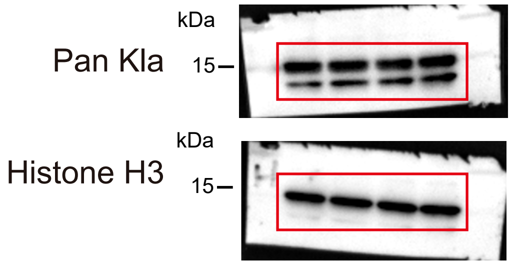

Figure S5I

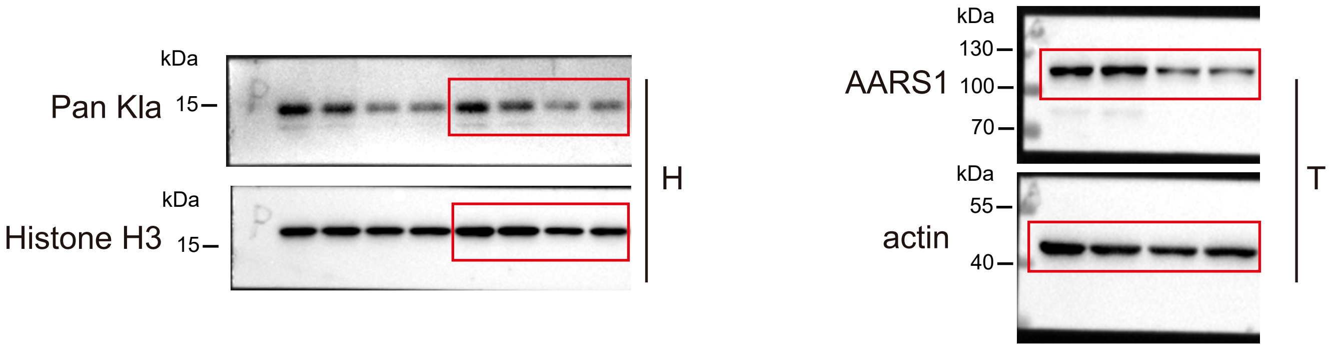

Figure S5J

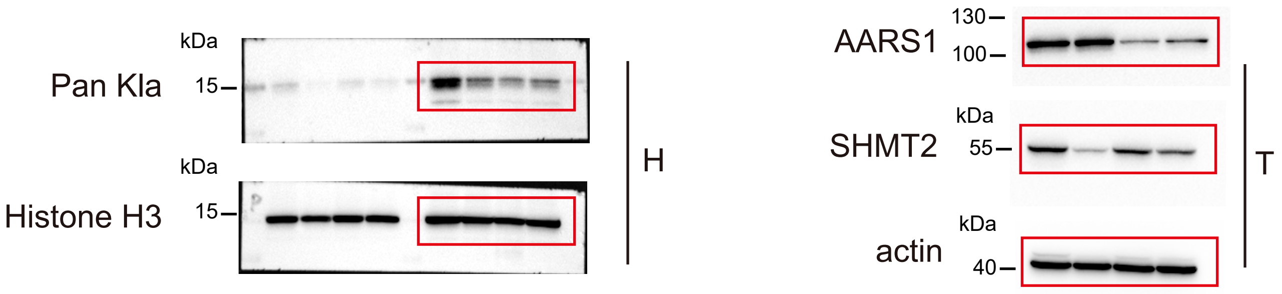

Figure S5K

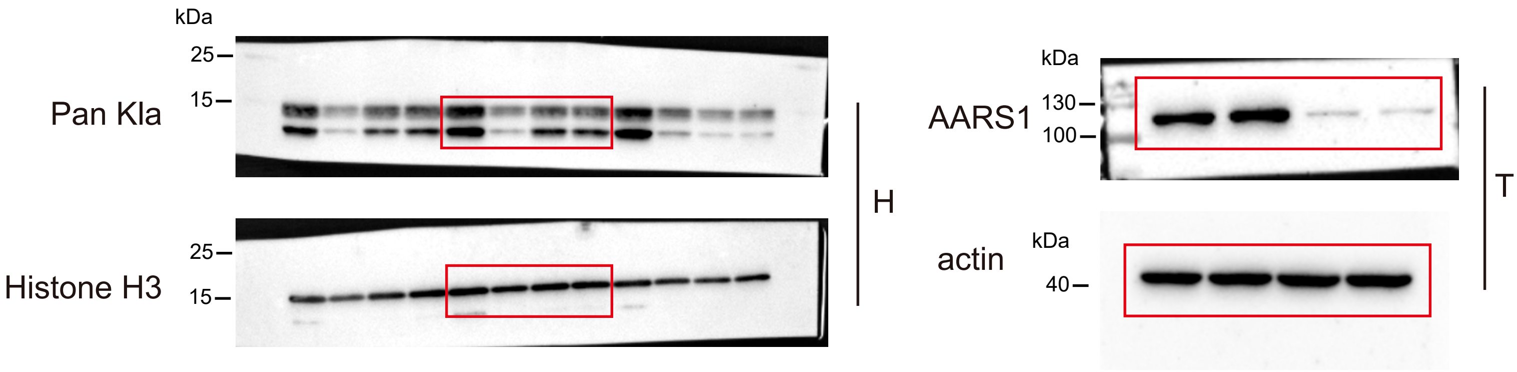

Full unedited blot/gel for Supplemental Figure 5

Figure S5L

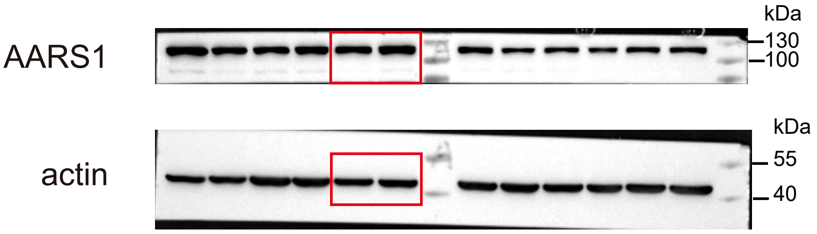

Figure S5M

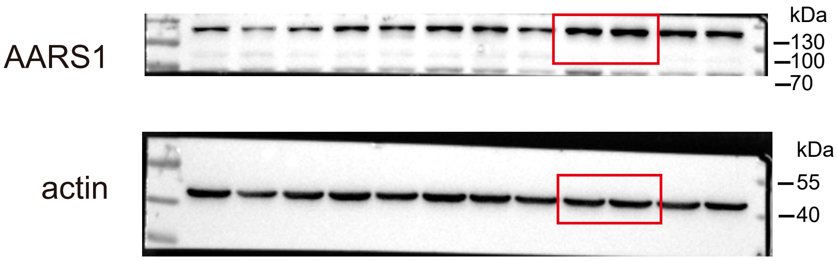

Figure S5N

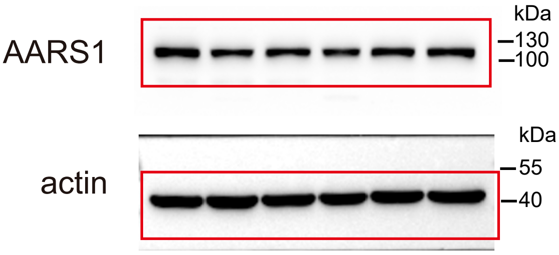

Figure S5O

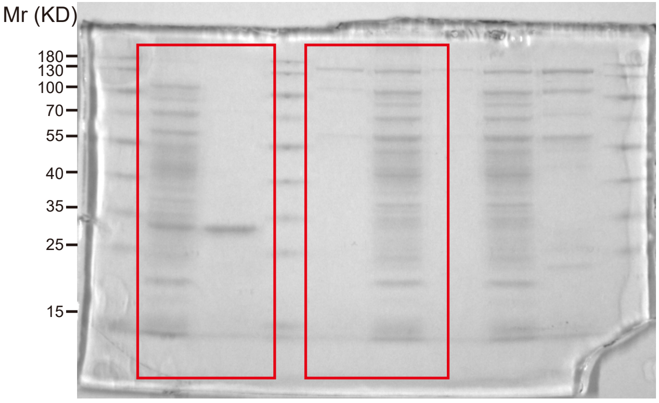

Supplement: Unedited blot and gel images [file jci-136-196094-s112.pdf]
